# Supplementary material for: Reappraisal of the extinct barbelthroat shark †Bavariscyllium and the nebulous origin of carcharhiniform galeomorphs
Source: Commun Biol. 2026 Feb 17;9:158. doi: 10.1038/s42003-025-09272-5 (PMC12913886; doi:10.1038/s42003-025-09272-5)
Supplement: Supplementary file 1 — Supplementary Information [file 42003_2025_9272_MOESM1_ESM.pdf]

SUPPLEMENTARY INFORMATION

Reappraisal of the extinct barbelthroat shark †*Bavariscyllium* and the nebulous origin of carcharhiniform galeomorphs

Sebastian Stumpf [1,2,*], Julia Türtscher [2], Faviel A. López-Romero [3], Eduardo Villalobos-Segura [2], Arnaud Begat [2,4], Manuel Amadori [2], Richard P. Dearden [5,6], Bruce Lauer [7], René Lauer [7], Andreas Hecker [8], & Jürgen Kriwet [2,4,*]

[1] Natural History Museum Vienna, Geological-Palaeontological Department, Burgring 7, 1010 Vienna, Austria

[2] University of Vienna, Department of Palaeontology, Geozentrum, Josef-Holaubek-Platz 2, 1090 Vienna, Austria

[3] Universidad Nacional Autónoma de México, Unidad de Sistemas Arrecifales, Instituto de Ciencias del Mar y Limnología, Puerto Morelos, Quintana Roo, México

[4] University of Vienna, Vienna Doctoral School of Ecology and Evolution (VDSEE), Djerassiplatz 1, 1030 Vienna, Austria

[5] Naturalis Biodiversity Center, Vertebrate Evolution, Development, and Ecology, Darwinweg 2, Leiden, The Netherlands

[6] University of Birmingham, School of Geography, Earth & Environmental Sciences, Edgbaston, Birmingham, B15 2TT, UK

[7] Lauer Foundation for Paleontology, Science and Education, Wheaton, Illinois, USA

[8] Jura-Museum Eichstätt, Burgstraße 19, 85072 Eichstätt, Germany

* Corresponding authors: sebastian.stumpf@univie.ac.at, juergen.kriwet@univie.ac.at

TABLE OF CONTENT

PART A. Morphometrics
PART B. Phylogenetic analysis
PART C. Additional information

Institutional abbreviations. AMNH, American Museum of Natural History, New York, USA; JME, Jura-Museum Eichstätt, Eichstätt, Germany; LACM, Los Angeles County Museum of Natural History, USA; LF, Lauer Foundation for Paleontology, Science and Education, Wheaton, Illinois, USA; MNHN, Museum national d'Histoire naturelle, Paris, France; NHMUK, Natural History Museum, London, UK; SIO, Scripps Institution of Oceanography, San Diego, USA; SMNK, Staatliches Museum für Naturkunde Karlsruhe, Germany; SMF, Senckenberg Naturmuseum, Frankfurt, Germany; SMNS, Staatliches Museum für Naturkunde Stuttgart, Germany; SNSB-BSPG, Bayerische Staatssammlung für Paläontologie und Geologie, Munich, Germany.

PART A. Morphometrics

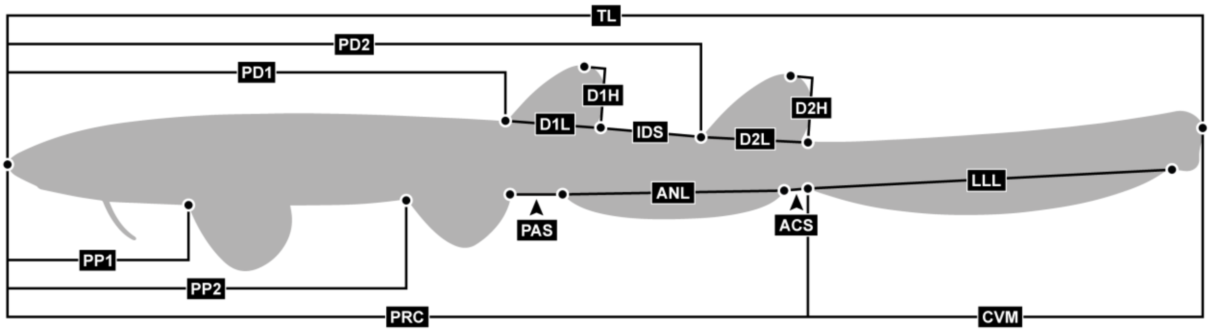

Supplementary Figure 1. Schematic diagram illustrating measurements used for the morphometric analysis. Abbreviations ([1]measurements adapted from Ebert et al. 2021; [2]newly added measurements): ACS[1], anal-caudal fin space; ANL[1], anal fin length; CVM[2], ventral caudal margin; D1H[1], first dorsal fin height; D2H[1], second dorsal fin height; D1L[1], first dorsal fin length; D2L[1], second dorsal fin length; IDS[1], interdorsal space; LLL[2], caudal fin lower lobe length; PAS[1], pelvic-anal fin space; PD1[1], pre-first dorsal fin length; PD2[1], pre-second dorsal fin length; PP1[1], Pre-pectoral fin length; PP2[1], pre-pelvic fin length; PRC[1], pre-caudal fin length; TL[1], total length.

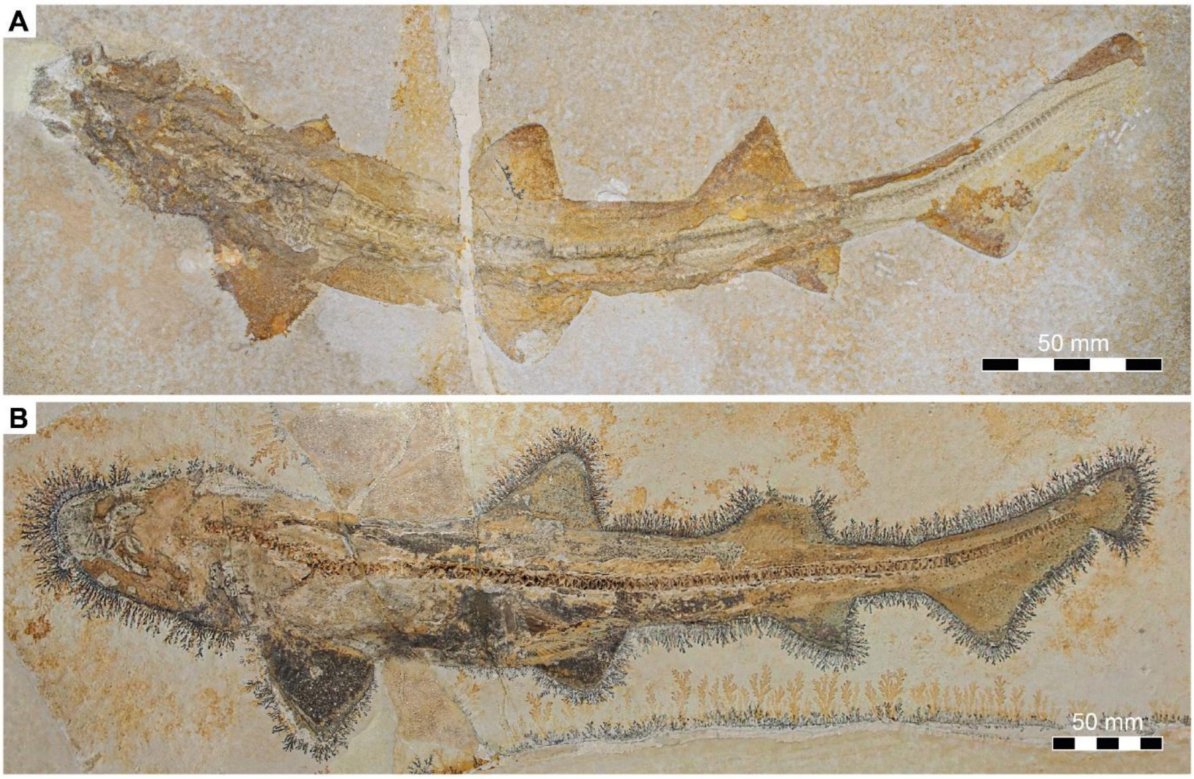

Supplementary Figure 2. Holomorphic specimens of †*Palaeoscyllium formosum* Wagner, 1857 from the Upper Jurassic Solnhofen Archipelago of southern Germany used for the morphometric analysis. **A**, SNSB-BSPG AS I 589, tooth-bearing specimen from the lower Tithonian of Solnhofen (teeth figured by Leidner & Thies 1999: fig. 3F; Kriwet & Klug 2004: fig. 15C, D; Thies & Leidner 2011: pls. 50, 51); **B**, SMNK-PAL 44950, tooth-bearing specimen from the lower Tithonian of Eichstätt.

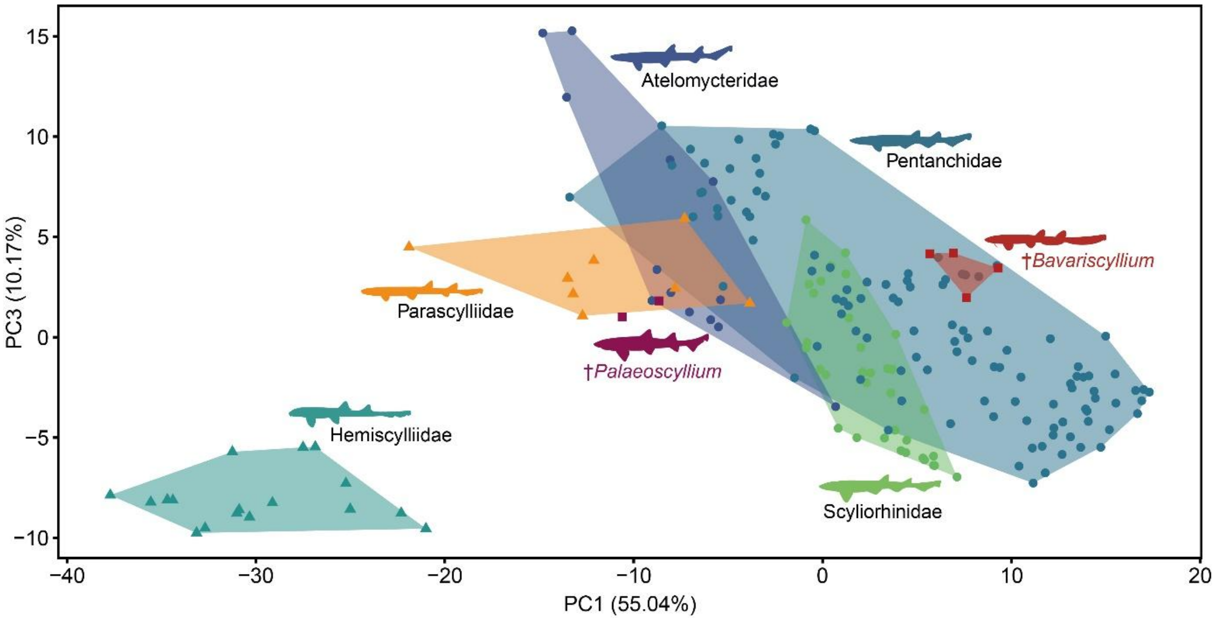

Supplementary Figure 3. Results of principal component analysis (PCA) plotting PC1 against PC3, including four †*Bavariscyllium tischlingeri* and two †*Palaeoscyllium formosum* specimens. Extant species aggregated at family level (Parascylliidae [*n* = 8]; Hemiscylliidae [*n* = 17]; Scyliorhinidae [*n* = 36]; Atelomycteridae [*n* = 13]; Pentanchidae [*n* = 86]), with orectolobiforms (*n* = 25) shown as triangles and carcharhiniforms (*n* = 160) as dots. Silhouettes of living taxa are based on Ebert et al. (2021).

Supplementary Table 1. PC axes generated from all measurements; the first three axes each explain more than 5% of the total variation.

	PC1	PC2	PC3	PC4	PC5	PC6	PC7	PC8	PC9	PC10	PC11	PC12	PC13	PC14	PC15
Standard deviation	12.1550	8.0766	5.2264	3.08821	2.36526	1.94885	1.77022	1.40156	1.26662	0.97275	0.87019	0.6288	0.5012	0.3494	0.23484
Proportion of Variance	0.5504	0.2430	0.1017	0.03553	0.02084	0.01415	0.01167	0.00732	0.00598	0.00352	0.00282	0.00147	0.00094	0.00045	0.00021
Cumulative Proportion	0.5504	0.7933	0.8951	0.93063	0.95147	0.96562	0.97729	0.98461	0.99059	0.99411	0.99693	0.9984	0.99934	0.99979	1.00000

Supplementary Table 2. Loadings of each measurement on the first three axes of the principal component analysis (PCA) and the percentage of variance (%var) explained by each.

**Measurement**		**PC1**	**PC2**	**PC3**
Pre-pectoral fin length	**PP1**	0.19912021	-0.02114964	-0.205721332
Pre-pelvic fin length	**PP2**	0.26527235	-0.488380246	-0.268778532
Pre-first dorsal fin length	**PD1**	0.24287694	-0.413943931	-0.20941546
Pre-second dorsal fin length	**PD2**	0.07373699	-0.394700149	0.106993338
Pre-caudal fin length	**PRC**	-0.31399657	-0.338458508	0.00419806
First dorsal fin length	**D1L**	-0.05769451	-0.002243173	-0.140673594
Second dorsal fin length	**D2L**	-0.04533027	0.059329653	-0.052467963
Anal fin length	**ANL**	0.17505379	0.23067051	-0.034507636
First dorsal fin height	**D1H**	-0.08545644	-0.041596155	-0.027990889
Second dorsal fin height	**D2H**	-0.07302574	0.017555663	0.001244956
Interdorsal space	**IDS**	-0.11476055	0.021847809	0.454217336
Caudal fin lower lobe length	**LLL**	0.27687299	0.328414165	-0.019055101
Ventral caudal margin	**CVM**	0.3097067	0.322131401	-0.009126467
Pelvic-anal fin space	**PAS**	-0.69747083	0.122473266	-0.376734427
Anal-caudal fin space	**ACS**	-0.07801413	-0.182952411	0.675951501
	**%var**	55.04%	24.30%	10.17%

Supplementary Table 3. Shapiro-Wilk normality test; bold indicates a *p*-value <0.05, indicating a non-normal distribution.

**Caudal fin lower lobe length**		**W**	***p***
Pre-pectoral fin length	**PP1**	0.9878	0.09984
Pre-pelvic fin length	**PP2**	0.98901	0.1485
Pre-first dorsal fin length	**PD1**	0.98435	**0.03182**
Pre-second dorsal fin length	**PD2**	0.99109	0.2869
Pre-caudal fin length	**PRC**	0.98374	**0.02605**
First dorsal fin length	**D1L**	0.98623	0.05932
Second dorsal fin length	**D2L**	0.99001	0.2053
Anal fin length	**ANL**	0.95176	**0.000004525**
First dorsal fin height	**D1H**	0.99318	0.5205
Second dorsal fin height	**D2H**	0.95151	**0.000004284**
Interdorsal space	**IDS**	0.97541	**0.001917**
Caudal fin lower lobe length	**LLL**	0.98134	**0.01198**
Ventral caudal margin	**CVM**	0.9864	0.06264
Pelvic-anal fin space	**PAS**	0.75549	**0.00000000000000022**
Anal-caudal fin space	**ACS**	0.90457	**0.00000000096**

Supplementary Table 4. Kruskal-Wallis rank sum test for non-normally distributed measurements across families; bold indicates a *p*-value <0.05.

**Measurement**		**df**	**χ2**	***p***
Pre-first dorsal fin length	**PD1**	6	90.613	**0.00000000000000022**
Pre-caudal fin length	**PRC**	6	87.207	**0.00000000000000022**
Anal fin length	**ANL**	6	92.995	**0.00000000000000022**
Second dorsal fin height	**D2H**	6	83.96	**0.0000000000000005421**
Interdorsal space	**IDS**	6	46.431	**0.0000000243**
Caudal fin lower lobe	**LLL**	6	84.178	**0.0000000000000004887**
Pelvic-anal fin space	**PAS**	6	101.24	**0.00000000000000022**
Anal-caudal fin space	**ACS**	6	51.381	**0.000000002484**

Supplementary Table 5. Post hoc pairiwse Wilcoxon test for non-normally distributed measurements across families; bold indicates a Bonferroni-corrected *p*-value <0.05.

**PD1**	Atelomycteridae	†*Bavariscyllium*	Hemiscylliidae	†*Palaeoscyllium*	Parascylliidae	Pentanchidae
Bavariscyllium	1.00000	-	-	-	-	-
Hemiscyllidae	**0.00018**	0.37193	-	-	-	-
Palaeoscyllium	0.40000	1.00000	1.00000	-	-	-
Parascylliidae	**0.01383**	0.08485	**0.000039**	0.93333	-	-
Pentanchidae	0.11952	0.05403	**0.0000000040**	0.33854	1.00000	-
Scyliorhinidae	**0.00000088**	**0.00046**	**0.0000000000013**	0.05974	0.80315	**0.00000050**

**PRC**	Atelomycteridae	†*Bavariscyllium*	Hemiscylliidae	†*Palaeoscyllium*	Parascylliidae	Pentanchidae
Bavariscyllium	**0.01765**	-	-	-	-	-
Hemiscyllidae	**0.0000042**	**0.00702**	-	-	-	-
Palaeoscyllium	1.00000	1.00000	1.00000	-	-	-
Parascylliidae	1.00000	0.08485	**0.01347**	1.00000	-	-
Pentanchidae	**0.00043**	**0.03231**	**0.0000000024**	1.00000	**0.02493**	-
Scyliorhinidae	0.34883	**0.00046**	**0.0000000000013**	1.00000	1.00000	**0.000043**

**ANL**	Atelomycteridae	†*Bavariscyllium*	Hemiscylliidae	†*Palaeoscyllium*	Parascylliidae	Pentanchidae
Bavariscyllium	**0.01765**	-	-	-	-	-
Hemiscyllidae	1.00000	**0.00702**	-	-	-	-
Palaeoscyllium	0.80000	1.00000	1.00000	-	-	-
Parascylliidae	0.16264	0.08485	1.00000	1.00000	-	-
Pentanchidae	**0.000092**	0.40168	**0.0000014**	0.40405	**0.00020**	-
Scyliorhinidae	1.00000	**0.00046**	1.00000	0.23898	**0.02990**	**0.0000000014**

**D2H**	Atelomycteridae	†*Bavariscyllium*	Hemiscylliidae	†*Palaeoscyllium*	Parascylliidae	Pentanchidae
Bavariscyllium	1.00000	-	-	-	-	-
Hemiscyllidae	1.00000	1.00000	-	-	-	-
Palaeoscyllium	1.00000	1.00000	1.00000	-	-	-
Parascylliidae	1.00000	1.00000	**0.01347**	0.93333	-	-
Pentanchidae	**0.000038**	0.06596	**0.000000024**	0.38108	**0.02493**	-
Scyliorhinidae	**0.000000022**	**0.00092**	**0.000000000016**	0.05974	**0.000045**	0.33302

**IDS**	Atelomycteridae	†*Bavariscyllium*	Hemiscylliidae	†*Palaeoscyllium*	Parascylliidae	Pentanchidae
Bavariscyllium	0.6706	-	-	-	-	-
Hemiscyllidae	**0.0024**	1.00000	-	-	-	-
Palaeoscyllium	1.00000	1.00000	0.9825	-	-	-
Parascylliidae	1.00000	1.00000	**0.0100**	1.00000	-	-
Pentanchidae	**0.0026**	1.00000	1.00000	1.00000	0.2945	-
Scyliorhinidae	**0.0000022**	0.1650	**0.0077**	0.5377	**0.000045**	**0.0012**

**LLL**	Atelomycteridae	†*Bavariscyllium*	Hemiscylliidae	†*Palaeoscyllium*	Parascylliidae	Pentanchidae
Bavariscyllium	**0.01765**	-	-	-	-	-
Hemiscyllidae	**0.000068**	**0.00702**	-	-	-	-
Palaeoscyllium	1.00000	1.00000	0.24561	-	-	-
Parascylliidae	1.00000	0.08485	**0.00528**	1.00000	-	-
Pentanchidae	**0.00577**	**0.01603**	**0.0000000037**	1.00000	0.11592	-
Scyliorhinidae	1.00000	**0.00046**	**0.0000000027**	1.00000	1.00000	**0.0000027**

**PAS**	Atelomycteridae	†*Bavariscyllium*	Hemiscylliidae	†*Palaeoscyllium*	Parascylliidae	Pentanchidae
Bavariscyllium	**0.0176**	-	-	-	-	-

Hemiscyllidae	**0.00000035**	**0.0070**	-	-	-	-
Palaeoscyllium	1.00000	1.00000	0.2456	-	-	-
Parascylliidae	0.0533	0.0848	**0.000039**	1.00000	-	-
Pentanchidae	**0.0000013**	1.00000	**0.00000000074**	0.3796	**0.000064**	-
Scyliorhinidae	**0.000000031**	1.00000	**0.0000000000013**	0.0597	**0.00000024**	**0.0028**

**ACS**	Atelomycteridae	†*Bavariscyllium*	Hemiscylliidae	†*Palaeoscyllium*	Parascylliidae	Pentanchidae
Bavariscyllium	**0.03529**	-	-	-	-	-
Hemiscyllidae	**0.0000076**	**0.00025**	-	-	-	-
Palaeoscyllium	1.00000	1.00000	**0.00083**	-	-	-
Parascylliidae	1.00000	0.08485	**0.000043**	1.00000	-	-
Pentanchidae	**0.03352**	1.00000	**0.000044**	1.00000	0.12765	-
Scyliorhinidae	**0.04196**	0.23668	**0.000000068**	1.00000	**0.00253**	1.00000

Supplementary Table 6. Results of the ANOVA testing for differences in normally distributed measurements across families; bold indicates a *p*-value <0.05.

**PP1**	**Df**	**SS**	**MS**	**Rsq**	**F**	**Z**	**Pr(>F)**
Family	6	689.84	114.973	0.3802	18.811	7.5081	**0.001**
Residuals	184	1124.59	6.112	0.6198			
Total	190	1814.43					

**PP2**	**Df**	**SS**	**MS**	**Rsq**	**F**	**Z**	**Pr(>F)**
Family	6	2688.2	448.03	0.46055	26.181	9.2563	**0.001**
Residuals	184	3148.7	17.11	0.53945			
Total	190	5836.9					

**PD2**	**Df**	**SS**	**MS**	**Rsq**	**F**	**Z**	**Pr(>F)**
Family	6	1327.5	221.25	0.49844	30.476	9.3735	**0.001**
Residuals	184	1335.8	7.26	0.50156			
Total	190	2663.4					

**D1L**	**Df**	**SS**	**MS**	**Rsq**	**F**	**Z**	**Pr(>F)**
Family	6	186.30	31.0502	0.33318	15.322	6.6542	**0.001**
Residuals	184	372.87	2.0264	0.66682			
Total	190	559.17					

**D2L**	**Df**	**SS**	**MS**	**Rsq**	**F**	**Z**	**Pr(>F)**
Family	6	171.06	28.5092	0.36296	17.473	7.8335	**0.001**
Residuals	184	300.22	1.6316	0.63704			
Total	190	471.27					

**D1H**	**Df**	**SS**	**MS**	**Rsq**	**F**	**Z**	**Pr(>F)**
Family	6	248.09	41.349	0.51627	32.73	9.885	**0.001**
Residuals	184	232.45	1.263	0.48373			
Total	190	480.55					

**CVM**	**Df**	**SS**	**MS**	**Rsq**	**F**	**Z**	**Pr(>F)**
Family	6	2025.1	337.52	0.49293	29.811	9.2118	**0.001**
Residuals	184	2083.2	11.32	0.50707			
Total	190	4108.3					

Supplementary Table 7. Results of the pairwise comparison to test for differences in the normally distributed measurements between the families; bold indicates a *p*-value <0.05.

**PP1**	**d**	**UCL (95%)**	**Z**	**Pr > d**
Atelomycteridae : †*Bavariscyllium*	1.45267527	3.398841	0.2568590	0.412
Atelomycteridae : Hemiscylliidae	2.14619266	2.07279	1.5542617	**0.046**
Atelomycteridae : †*Palaeoscyllium*	2.23427989	4.68141	0.4214411	0.355
Atelomycteridae : Parascylliidae	1.54619970	2.723005	0.6537077	0.276
Atelomycteridae : Pentanchidae	3.31776664	1.710230	2.9868273	**0.001**
Atelomycteridae : Scyliorhinidae	2.46013652	1.873532	2.1220197	**0.014**
†*Bavariscyllium* : Hemiscyllidae	0.69351739	3.294577	-0.5626352	0.707
†*Bavariscyllium* : †*Palaeoscyllium*	3.68695516	5.238125	0.9500016	0.181
†*Bavariscyllium* : Parascylliidae	0.09352443	3.635137	-1.7725560	0.960
†*Bavariscyllium* : Pentanchidae	4.77044190	2.992760	2.4227692	**0.004**
†*Bavariscyllium* : Scyliorhinidae	3.91281179	3.209511	1.9756160	**0.017**
Hemiscylliidae : †*Palaeoscyllium*	4.38047255	4.598740	1.5007856	0.064
Hemiscylliidae : Parascylliidae	0.59999296	2.578212	-0.4746781	0.674
Hemiscylliidae : Pentanchidae	5.46395930	1.636873	4.3129285	**0.001**
Hemiscylliidae : Scyliorhinidae	4.60632918	1.857172	3.7138596	**0.001**
†*Palaeoscyllium* : Parascylliidae	3.78047959	4.912021	1.1445275	0.136
†*Palaeoscyllium* : Pentanchidae	1.08348674	4.280821	-0.3655641	0.645
†*Palaeoscyllium* : Scyliorhinidae	0.22585663	4.504118	-1.5142676	0.937
Parascylliidae : Pentanchidae	4.86396634	2.288249	3.1759602	**0.001**
Parascylliidae : Scyliorhinidae	4.00633622	2.388814	2.5322137	**0.002**
Pentanchidae : Scyliorhinidae	0.85763011	1.089156	1.1200701	0.140

**PP2**	**d**	**UCL (95%)**	**Z**	**Pr > d**
Atelomycteridae : †*Bavariscyllium*	6.5706971	6.399271	1.62727437	**0.042**
Atelomycteridae : Hemiscylliidae	7.2362958	4.053049	2.86977699	**0.002**
Atelomycteridae : †*Palaeoscyllium*	2.8075028	8.49634	-0.01008756	0.509
Atelomycteridae : Parascylliidae	3.7028632	4.922014	1.14041357	0.138
Atelomycteridae : Pentanchidae	2.2514273	3.114301	1.03163109	0.153
Atelomycteridae : Scyliorhinidae	6.2712697	3.498380	2.79301382	**0.001**
†*Bavariscyllium* : Hemiscyllidae	0.6655987	6.475660	-1.06215734	0.839
†*Bavariscyllium* : †*Palaeoscyllium*	9.3781998	9.542022	1.56655068	0.057
†*Bavariscyllium* : Parascylliidae	2.8678339	6.799770	0.32435251	0.407
†*Bavariscyllium* : Pentanchidae	8.8221244	5.738812	2.45843803	**0.001**
†*Bavariscyllium* : Scyliorhinidae	12.8419667	5.957049	3.29276401	**0.001**
Hemiscylliidae : †*Palaeoscyllium*	10.0437986	8.245305	1.99453553	**0.014**
Hemiscylliidae : Parascylliidae	3.5334326	4.508124	1.12419881	0.133
Hemiscylliidae : Pentanchidae	9.4877231	2.910943	4.27684618	**0.001**
Hemiscylliidae : Scyliorhinidae	13.5075654	3.199170	4.93678057	**0.001**
†*Palaeoscyllium* : Parascylliidae	6.5103660	8.768267	1.07292716	0.156
†*Palaeoscyllium* : Pentanchidae	0.5560754	8.070567	-1.28390660	0.884
†*Palaeoscyllium* : Scyliorhinidae	3.4637669	7.925049	0.29795261	0.407
Parascylliidae : Pentanchidae	5.9542905	3.754652	2.42346571	**0.006**
Parascylliidae : Scyliorhinidae	9.9741329	4.014173	3.31084832	**0.001**
Pentanchidae : Scyliorhinidae	4.0198423	2.065733	2.91209497	**0.001**

**PD2**	**d**	**UCL (95%)**	**Z**	**Pr > d**
Atelomycteridae : †*Bavariscyllium*	6.1958424	4.100053	2.33434523	**0.005**
Atelomycteridae : Hemiscylliidae	7.5680039	2.79457	3.69578075	**0.001**
Atelomycteridae : †*Palaeoscyllium*	6.0183790	5.754557	1.68440886	**0.032**
Atelomycteridae : Parascylliidae	2.7727535	3.241412	1.30548386	0.095

Atelomycteridae : Pentanchidae	1.3643692	2.139396	0.83479898	0.224
Atelomycteridae : Scyliorhinidae	1.8909499	2.368379	1.22378118	0.112
†*Bavariscyllium* : Hemiscyllidae	1.3721615	4.279232	0.06083868	0.481
†*Bavariscyllium* : †*Palaeoscyllium*	0.1774633	6.701707	-1.75961498	0.962
†*Bavariscyllium* : Parascylliidae	8.9685959	4.380866	3.07276077	**0.001**
†*Bavariscyllium* : Pentanchidae	4.8314731	3.688784	2.07635754	**0.012**
†*Bavariscyllium* : Scyliorhinidae	8.0867923	4.004631	2.99368085	**0.001**
Hemiscylliidae : †*Palaeoscyllium*	1.5496248	5.578222	-0.19946448	0.593
Hemiscylliidae : Parascylliidae	10.3407574	3.160479	4.33128369	**0.001**
Hemiscylliidae : Pentanchidae	6.2036346	1.950052	4.27794565	**0.001**
Hemiscylliidae : Scyliorhinidae	9.4589538	2.153916	4.91582193	**0.001**
†*Palaeoscyllium* : Parascylliidae	8.7911325	5.769457	2.38950837	**0.002**
†*Palaeoscyllium* : Pentanchidae	4.6540098	5.413236	1.34940593	0.094
†*Palaeoscyllium* : Scyliorhinidae	7.9093289	5.509348	2.32028365	**0.004**
Parascylliidae : Pentanchidae	4.1371227	2.654416	2.47789497	**0.004**
Parascylliidae : Scyliorhinidae	0.8818036	2.979853	-0.12225075	0.556
Pentanchidae : Scyliorhinidae	3.2553191	1.466518	3.31413946	**0.001**

**D1L**	**d**	**UCL (95%)**	**Z**	**Pr > d**
Atelomycteridae : †*Bavariscyllium*	0.65468241	1.9615703	-0.00912071	0.525
Atelomycteridae : Hemiscylliidae	2.45194680	1.2240922	3.06327273	**0.001**
Atelomycteridae : †*Palaeoscyllium*	1.68117460	2.6241562	0.91820163	0.182
Atelomycteridae : Parascylliidae	1.00129340	1.5883059	0.88662889	0.203
Atelomycteridae : Pentanchidae	0.75236877	0.953983	1.17981506	0.125
Atelomycteridae : Scyliorhinidae	0.53967166	1.0477404	0.56862937	0.308
†*Bavariscyllium* : Hemiscyllidae	3.10662920	1.8452844	2.55804244	**0.003**
†*Bavariscyllium* : †*Palaeoscyllium*	2.33585701	2.8207792	1.24021621	0.108
†*Bavariscyllium* : Parascylliidae	0.34661099	2.1094455	-0.63247279	0.721
†*Bavariscyllium* : Pentanchidae	0.09768636	1.7179579	-1.43559044	0.905
†*Bavariscyllium* : Scyliorhinidae	1.19435407	1.7821894	0.93556619	0.184
Hemiscylliidae : †*Palaeoscyllium*	0.77077220	2.5185898	-0.02055044	0.514
Hemiscylliidae : Parascylliidae	3.45324020	1.5058075	3.31848263	**0.001**
Hemiscylliidae : Pentanchidae	3.20431556	0.8688010	4.69728695	**0.001**
Hemiscylliidae : Scyliorhinidae	1.91227514	0.9810523	2.97476928	**0.001**
†*Palaeoscyllium* : Parascylliidae	2.68246800	2.5941116	1.63525864	**0.046**
†*Palaeoscyllium* : Pentanchidae	2.43354337	2.4481292	1.59295398	0.054
†*Palaeoscyllium* : Scyliorhinidae	1.14150294	2.5160054	0.45933596	0.324
Parascylliidae : Pentanchidae	0.24892463	1.2803776	-0.50101670	0.699
Parascylliidae : Scyliorhinidae	1.54096506	1.3629065	1.84725298	**0.021**
Pentanchidae : Scyliorhinidae	1.29204043	0.6297794	3.14636843	**0.001**

**D2L**	**d**	**UCL (95%)**	**Z**	**Pr > d**
Atelomycteridae : †*Bavariscyllium*	1.1341414	1.7192049	0.8645701	0.218
Atelomycteridae : Hemiscylliidae	1.1654489	1.1528398	1.6230938	**0.043**
Atelomycteridae : †*Palaeoscyllium*	0.6279245	2.3758253	-0.2588193	0.613
Atelomycteridae : Parascylliidae	1.3684573	1.3686005	1.5688713	0.051
Atelomycteridae : Pentanchidae	0.9553217	0.9136806	1.6771524	**0.041**
Atelomycteridae : Scyliorhinidae	2.4115788	0.9568643	3.6042929	**0.001**
†*Bavariscyllium* : Hemiscyllidae	2.2995903	1.7150735	2.1256565	**0.011**
†*Bavariscyllium* : †*Palaeoscyllium*	1.7620659	2.6915125	0.8779703	0.202
†*Bavariscyllium* : Parascylliidae	0.2343159	1.9260208	-0.9677428	0.817
†*Bavariscyllium* : Pentanchidae	0.1788197	1.5232854	-1.0304306	0.833
†*Bavariscyllium* : Scyliorhinidae	1.2774374	1.6170136	1.1841287	0.125
Hemiscylliidae : †*Palaeoscyllium*	0.5375244	2.2541921	-0.3874814	0.647
Hemiscylliidae : Parascylliidae	2.5339062	1.2889877	3.0863125	**0.001**

Hemiscylliidae : Pentanchidae	2.1207706	0.7648338	3.7962224	**0.001**
Hemiscylliidae : Scyliorhinidae	3.5770277	0.8801739	4.9369232	**0.001**
†*Palaeoscyllium* : Parascylliidae	1.9963818	2.4484286	1.2812917	0.107
†*Palaeoscyllium* : Pentanchidae	1.5832462	2.2511761	1.0146353	0.166
†*Palaeoscyllium* : Scyliorhinidae	3.0395033	2.2697427	2.1298133	**0.010**
Parascylliidae : Pentanchidae	0.4131356	1.1543461	0.1388712	0.450
Parascylliidae : Scyliorhinidae	1.0431215	1.1877777	1.3418441	0.091
Pentanchidae : Scyliorhinidae	1.4562571	0.5851981	3.5269387	**0.001**

**D1H**	**d**	**UCL (95%)**	**Z**	**Pr > d**
Atelomycteridae : †*Bavariscyllium*	0.8139173	1.7466413	0.34943959	0.391
Atelomycteridae : Hemiscylliidae	0.9475064	1.0351321	1.42308430	0.076
Atelomycteridae : †*Palaeoscyllium*	0.6907640	2.3093996	-0.22010179	0.582
Atelomycteridae : Parascylliidae	1.0875448	1.3204888	1.22884043	0.116
Atelomycteridae : Pentanchidae	2.2389467	0.8588647	3.69857845	**0.001**
Atelomycteridae : Scyliorhinidae	0.3367498	0.9577704	0.08327708	0.480
†*Bavariscyllium* : Hemiscyllidae	1.7614237	1.6643835	1.67851081	**0.038**
†*Bavariscyllium* : †*Palaeoscyllium*	1.5046813	2.635728	0.62536292	0.300
†*Bavariscyllium* : Parascylliidae	0.2736275	1.8251680	-0.72529462	0.747
†*Bavariscyllium* : Pentanchidae	1.4250294	1.5239507	1.44222035	0.073
†*Bavariscyllium* : Scyliorhinidae	0.4771674	1.6041303	-0.14987261	0.568
Hemiscylliidae : †*Palaeoscyllium*	0.2567424	2.2274652	-1.09698992	0.840
Hemiscylliidae : Parascylliidae	2.0350512	1.2578662	2.57587981	**0.002**
Hemiscylliidae : Pentanchidae	3.1864531	0.7963615	4.99077246	**0.001**
Hemiscylliidae : Scyliorhinidae	1.2842562	0.8983583	2.35014408	**0.003**
†*Palaeoscyllium* : Parascylliidae	1.7783088	2.3686197	1.05650101	0.156
†*Palaeoscyllium* : Pentanchidae	2.9297108	2.2009588	2.21643476	**0.003**
†*Palaeoscyllium* : Scyliorhinidae	1.0275139	2.2317846	0.31465042	0.417
Parascylliidae : Pentanchidae	1.1514019	1.0665951	1.73825024	**0.030**
Parascylliidae : Scyliorhinidae	0.7507950	1.1489617	0.88101880	0.204
Pentanchidae : Scyliorhinidae	1.9021969	0.5495389	4.49647065	**0.001**

**CVM**	**d**	**UCL (95%)**	**Z**	**Pr > d**
Atelomycteridae : †*Bavariscyllium*	12.4285342	5.173022	3.6208440	**0.001**
Atelomycteridae : Hemiscylliidae	4.4623926	3.34472	2.1481370	**0.008**
Atelomycteridae : †*Palaeoscyllium*	1.4239344	6.812374	-0.6490771	0.734
Atelomycteridae : Parascylliidae	0.3886792	4.147615	-1.0940484	0.850
Atelomycteridae : Pentanchidae	5.0046995	2.550155	2.9841008	**0.001**
Atelomycteridae : Scyliorhinidae	1.6640744	2.742214	0.7253649	0.270
†*Bavariscyllium* : Hemiscyllidae	16.8909269	5.022184	4.5523447	**0.001**
†*Bavariscyllium* : †*Palaeoscyllium*	11.0045999	7.537219	2.3400946	**0.004**
†*Bavariscyllium* : Parascylliidae	12.8172134	5.485576	3.4677980	**0.001**
†*Bavariscyllium* : Pentanchidae	7.4238348	4.503307	2.6157291	**0.001**
†*Bavariscyllium* : Scyliorhinidae	10.7644598	4.776290	3.4138178	**0.001**
Hemiscylliidae : †*Palaeoscyllium*	5.8863270	6.664619	1.3287923	0.095
Hemiscylliidae : Parascylliidae	4.0737134	4.069868	1.5982486	0.050
Hemiscylliidae : Pentanchidae	9.4670921	2.419401	4.8748855	**0.001**
Hemiscylliidae : Scyliorhinidae	6.1264671	2.883273	3.1015324	**0.001**
†*Palaeoscyllium* : Parascylliidae	1.8126136	7.073991	-0.3466955	0.647
†*Palaeoscyllium* : Pentanchidae	3.5807651	6.601989	0.6106208	0.285
†*Palaeoscyllium* : Scyliorhinidae	0.2401401	6.474592	-1.7492757	0.956
Parascylliidae : Pentanchidae	5.3933787	3.351263	2.4854703	**0.006**
Parascylliidae : Scyliorhinidae	2.0527536	3.701342	0.6694530	0.273
Pentanchidae : Scyliorhinidae	3.3406250	1.703095	2.9151744	**0.001**

Supplementary Table 8. Kruskal-Wallis rank sum test on non-normally distributed measurements across orders; bold indicates a *p*-value <0.05.

**Measurement**		**df**	**χ2**	***p***
Pre-first dorsal fin length	**PD1**	3	34.353	**0.0000001669**
Pre-caudal fin length	**PRC**	3	55.781	**0.000000000004678**
Anal fin length	**ANL**	3	42.779	**0.000000002741**
Second dorsal fin height	**D2H**	3	52.729	**0.00000000002094**
Interdorsal space	**IDS**	3	5.1101	0.1639
caudal fin lower lobe length	**LLL**	3	50.269	**0.00000000007001**
Pelvic-anal fin space	**PAS**	3	67.709	**0.0000000000000132**
Anal-caudal fin space	**ACS**	3	9.4914	**0.02342**

Supplementary Table 9. Post hoc pairiwse Wilcoxon test for non-normally distributed measurements across orders; bold indicates a Bonferroni-corrected *p*-value <0.05.

**PD1**	†*Bavariscyllium*	Carcharhiniformes	Orectolobiformes
Carcharhiniformes	**0.014**	-	-
Orectolobiformes	1.000	**0.000016**	-
Palaeoscyllium	0.800	0.093	1.000

**PRC**	†*Bavariscyllium*	Carcharhiniformes	Orectolobiformes
Carcharhiniformes	**0.00676**	-	-
Orectolobiformes	**0.00051**	**0.000000000068**	-
Palaeoscyllium	0.80000	1.00000	1.00000

**ANL**	†*Bavariscyllium*	Carcharhiniformes	Orectolobiformes
Carcharhiniformes	**0.04404**	-	-
Orectolobiformes	**0.00051**	**0.00000013**	-
Palaeoscyllium	0.80000	0.11939	0.68376

**D2H**	†*Bavariscyllium*	Carcharhiniformes	Orectolobiformes
Carcharhiniformes	**0.03**	-	-
Orectolobiformes	1.00	**0.0000000004**	-
Palaeoscyllium	0.80	0.13	1.00

**IDS**	†*Bavariscyllium*	Carcharhiniformes	Orectolobiformes
Carcharhiniformes	1.00	-	-
Orectolobiformes	1.00	0.53	-
Palaeoscyllium	0.80	1.00	1.00

**LLL**	†*Bavariscyllium*	Carcharhiniformes	Orectolobiformes
Carcharhiniformes	**0.00411**	-	-
Orectolobiformes	**0.00051**	**0.000000002**	-
Palaeoscyllium	0.80000	1.00000	0.85470

**PAS**	†*Bavariscyllium*	Carcharhiniformes	Orectolobiformes
Carcharhiniformes	1.00000	-	-
Orectolobiformes	**0.00051**	**0.0000000000000098**	-
Palaeoscyllium	0.80000	0.14560	0.13675

**ACS**	†*Bavariscyllium*	Carcharhiniformes	Orectolobiformes
Carcharhiniformes	1.000	-	-
Orectolobiformes	1.000	**0.031**	-
Palaeoscyllium	0.800	1.000	1.000

Supplementary Table 10. Results of the ANOVA testing for differences in normally distributed measurements across orders; bold indicates a *p*-value <0.05.

**PP1**	**Df**	**SS**	**MS**	**Rsq**	**F**	**Z**	**Pr(>F)**
Order	3	552.53	184.178	0.30452	27.293	6.5285	**0.001**
Residuals	187	1261.90	6.748	0.69548			
Total	190	1814.43					

**PP2**	**Df**	**SS**	**MS**	**Rsq**	**F**	**Z**	**Pr(>F)**
Order	3	2055.9	685.30	0.35223	33.894	7.4686	**0.001**
Residuals	187	3781.0	20.22	0.64777			
Total	190	5836.9					

**PD2**	**Df**	**SS**	**MS**	**Rsq**	**F**	**Z**	**Pr(>F)**
Order	3	453.92	151.306	0.17043	12.806	4.5866	**0.001**
Residuals	187	2209.44	11.815	0.82957			
Total	190	2663.36					

**D1L**	**Df**	**SS**	**MS**	**Rsq**	**F**	**Z**	**Pr(>F)**
Order	3	73.78	24.5936	0.13195	9.4749	3.7977	**0.001**
Residuals	187	485.39	2.5956	0.86805			
Total	190	559.17					

**D2L**	**Df**	**SS**	**MS**	**Rsq**	**F**	**Z**	**Pr(>F)**
Order	3	57.92	19.3069	0.1229	8.7344	3.6665	**0.001**
Residuals	187	413.35	2.2104	0.8771			
Total	190	471.27					

**D1H**	**Df**	**SS**	**MS**	**Rsq**	**F**	**Z**	**Pr(>F)**
Order	3	89.65	29.8848	0.18657	14.297	5.754	**0.001**
Residuals	187	390.89	2.0903	0.81343			
Total	190	480.55					

**CVM**	**Df**	**SS**	**MS**	**Rsq**	**F**	**Z**	**Pr(>F)**
Order	3	1422.1	474.04	0.34616	33	7.4797	**0.001**
Residuals	187	2686.2	14.36	0.65384			
Total	190	4108.3					

Supplementary Table 11. Results of the pairwise comparison testing for differences in normally distributed measurements between orders; bold indicates a *p*-value <0.05.

**PP1**	**d**	**UCL (95%)**	**Z**	**Pr > d**
†*Bavariscyllium* : Carcharhiniformes	4.3079066	2.995118	2.2172742	**0.010**
†*Bavariscyllium* : Orectolobiformes	0.5015196	3.192293	-0.8119694	0.793
†*Bavariscyllium* : †*Palaeoscyllium*	3.6869552	5.238125	0.9500016	0.181
Carcharhiniformes : Orectolobiformes	4.8094262	1.4038	4.4126934	**0.001**
Carcharhiniformes : †*Palaeoscyllium*	0.6209514	4.281456	-0.8545570	0.794
Orectolobiformes : †*Palaeoscyllium*	4.1884748	4.46882	1.4347234	0.068

**PP2**	**d**	**UCL (95%)**	**Z**	**Pr > d**
†*Bavariscyllium* : Carcharhiniformes	9.5436604	5.743044	2.629376	**0.001**
†*Bavariscyllium* : Orectolobiformes	0.4650997	6.173204	-1.250965	0.878
†*Bavariscyllium* : †*Palaeoscyllium*	9.3781998	9.542022	1.566551	0.057
Carcharhiniformes : Orectolobiformes	9.0785607	2.383396	4.779387	**0.001**
Carcharhiniformes : †*Palaeoscyllium*	0.1654606	7.972625	-1.800091	0.959
Orectolobiformes : †*Palaeoscyllium*	8.9131001	8.31088	1.761340	**0.030**

**PD2**	**d**	**UCL (95%)**	**Z**	**Pr > d**
†*Bavariscyllium* : Carcharhiniformes	5.6747749	3.632858	2.38763722	**0.006**
†*Bavariscyllium* : Orectolobiformes	1.9368809	3.985692	0.49760130	0.327
†*Bavariscyllium* : †*Palaeoscyllium*	0.1774633	6.701707	-1.75961498	0.962
Carcharhiniformes : Orectolobiformes	3.7378941	1.512409	3.49463718	**0.001**
Carcharhiniformes : †*Palaeoscyllium*	5.4973116	5.399446	1.64921440	**0.047**
Orectolobiformes : †*Palaeoscyllium*	1.7594175	5.496879	-0.05753328	0.540

**D1L**	**d**	**UCL (95%)**	**Z**	**Pr > d**
†*Bavariscyllium* : Carcharhiniformes	0.2541527	1.7377287	-0.7775161	0.765
†*Bavariscyllium* : Orectolobiformes	2.0015923	1.7959587	1.7433765	**0.034**
†*Bavariscyllium* : †*Palaeoscyllium*	2.3358570	2.8207792	1.2402162	0.108
Carcharhiniformes : Orectolobiformes	1.7474396	0.7111476	3.6246479	**0.001**
Carcharhiniformes : †*Palaeoscyllium*	2.0817043	2.4667465	1.3474619	0.098
Orectolobiformes : †*Palaeoscyllium*	0.3342647	2.4649943	-0.7613846	0.769

**D2L**	**d**	**UCL (95%)**	**Z**	**Pr > d**
†*Bavariscyllium* : Carcharhiniformes	0.07121827	1.5180331	-1.5920549	0.931
†*Bavariscyllium* : Orectolobiformes	1.48874030	1.6743821	1.3870741	0.080
†*Bavariscyllium* : †*Palaeoscyllium*	1.76206587	2.6915125	0.8779703	0.202
Carcharhiniformes : Orectolobiformes	1.55995856	0.6330282	3.5751664	**0.001**
Carcharhiniformes : †*Palaeoscyllium*	1.83328414	2.2656151	1.2486773	0.105
Orectolobiformes : †*Palaeoscyllium*	0.27332557	2.280921	-0.9448340	0.817

**D1H**	**d**	**UCL (95%)**	**Z**	**Pr > d**
†*Bavariscyllium* : Carcharhiniformes	0.8151207	1.5102001	0.5407749	0.315
†*Bavariscyllium* : Orectolobiformes	1.1102073	1.6156018	0.9505447	0.195
†*Bavariscyllium* : †*Palaeoscyllium*	1.5046813	2.635728	0.6253629	0.300
Carcharhiniformes : Orectolobiformes	1.9253280	0.6668443	4.2628377	**0.001**
Carcharhiniformes : †*Palaeoscyllium*	2.3198020	2.1985255	1.7059108	**0.032**
Orectolobiformes : †*Palaeoscyllium*	0.3944740	2.1627433	-0.7446598	0.764

**CVM**	**d**	**UCL (95%)**	**Z**	**Pr > d**
†*Bavariscyllium* : Carcharhiniformes	8.582107	4.497966	2.97550740	**0.001**

†*Bavariscyllium* : Orectolobiformes	15.587339	4.698615	4.50023447	**0.001**
†*Bavariscyllium* : †*Palaeoscyllium*	11.004600	7.537219	2.34009461	**0.004**
Carcharhiniformes : Orectolobiformes	7.005231	2.002662	4.39094803	**0.001**
Carcharhiniformes : †*Palaeoscyllium*	2.422493	6.427614	0.07415724	0.496
Orectolobiformes : †*Palaeoscyllium*	4.582739	6.560848	0.94687693	0.186

Supplementary Table 12. Linear discriminant analysis (LDA) classification table, with extant species aggregated at family level; the number of specimens/species in each group classified (columns) against the actual group (rows).

actual	classified<br>**Atelomycteridae**	***†Bavariscyllium***	**Hemiscylliidae**	***†Palaeoscyllium***	**Parascylliidae**	**Pentanchidae**	**Scyliorhinidae**
**Atelomycteridae**	9	0	0	0	0	4	0
†Bavariscyllium	0	4	0	0	0	0	0
**Hemiscylliidae**	0	0	17	0	0	0	0
†Palaeoscyllium	0	0	0	2	0	0	0
**Parascylliidae**	0	0	0	0	8	0	0
**Pentanchidae**	3	0	0	0	1	106	1
**Scyliorhinidae**	0	0	0	0	0	4	32

Supplementary Table 13. Linear discriminant analysis (LDA) classification table, with extant species aggregated at order level; the number of specimens/species in each group classified (columns) against the actual group (rows).

	classified			
actual	**Carcharhiniformes**	**†*Bavariscyllium***	**†*Palaeoscyllium***	**Orectolobiformes**
**Carcharhiniformes**	159	0	1	0
**†*Bavariscyllium***	0	4	0	0
**†*Palaeoscyllium***	2	0	0	0
**Orectolobiformes**	4	0	0	21

Supplementary Table 14. Disparity across groups as sum of variances, with extant species aggregated at family level. Subsets indicate number of specimens/species, observed disparity, bootstrapped median disparity and pairwise comparison *p*-value; bold indicates a *p*-value <0.05.

Subsets	n	obs	bs.median	Atelomycteridae	†*Bavariscyllium*	Hemiscylliidae	†*Palaeoscyllium*	Parascylliidae	Pentanchidae	Scyliorhinidae
Atelomycteridae	13	89.9	83.6							
†*Bavariscyllium*	4	16.4	12.8	**51.9e-33**						
Hemiscylliidae	17	57.4	53.5	**6.71e-27**	**5.19e-33**					
†*Palaeoscyllium*	2	55.2	0	**2.77e-30**	1	**5.68e-06**				
Parascylliidae	8	61.1	46.0	**2.45e-23**	**2.68e-32**	2.62e-01	**3.44e-04**			
Pentanchidae	111	157.6	156.1	**5.38e-33**	**5.19e-33**	**5.38e-33**	**4.71e-34**	**5.37e-33**		
Scyliorhinidae	36	58.6	55.7	**6.96e-26**	**5.19e-33**	6.82e-02	**3.77e-11**	**4.89e-04**	**5.38e-33**	

Supplementary Table 15. Disparity across groups as sum of variances, with extant species aggregated at order level. Subsets indicate the number of specimens/species, observed disparity, bootstrapped median disparity and pairwise comparison *p*-value; bold indicates a *p*-value <0.05.

Subsets	n	obs	bs.median	Carcharhiniformes	†*Bavariscyllium*	Orectolobiformes	†*Palaeoscyllium*
Carcharhiniformes	160	163.4	163.4				
†*Bavariscyllium*	4	16.4	12.2	**1.5e-33**			
Orectolobiforms	25	200.4	193.4	**4.08e-16**	**1.5e-33**		
†*Palaeoscyllium*	2	55.2	0.0	**1.36e-34**	1	**1.36e-34**	

Supplementary Table 16. Disparity across groups as sum of ranges, with extant species aggregated at family level. Subsets indicate number of specimens/species, observed disparity, bootstrapped median disparity and pairwise comparison *p*-value; bold indicates a *p*-value <0.05.

Subsets	n	obs	bs.median	Atelomycteridae	†*Bavariscyllium*	Hemiscylliidae	†*Palaeoscyllium*	Parascylliidae	Pentanchidae	Scyliorhinidae
Atelomycteridae	13	26.27	23.15	-						
†*Bavariscyllium*	4	9.22	6.52	**4.76E-33**	-					
Hemiscylliidae	17	25.7	22.27	1.02E-01	**4.77E-33**	-				
†*Palaeoscyllium*	2	9.02	9.02	**4.76E-34**	1	**4.76E-34**				
Parascylliidae	8	17.16	14.52	**5.36E-33**	**6.97E-32**	**5.36E-33**	**1.08E-32**			
Pentanchidae	111	49.89	46.79	**5.37E-33**	**4.77E-33**	**5.38E-33**	**4.76E-34**	**5.36E-33**		
Scyliorhinidae	36	27.57	25.69	**1.06E-15**	**4.77E-33**	**5.51E-20**	**4.76E-34**	**5.36E-33**	**5.38E-33**	-

Supplementary Table 17. Disparity across groups as sum of ranges, with extant species aggregated at order level. Subsets indicate number of specimens/species, observed disparity, bootstrapped median disparity and pairwise comparison *p*-value; bold indicates a *p*-value <0.05.

Subsets	n	obs	bs.median	Carcharhiniformes	†*Bavariscyllium*	Orectolobiformes	†*Palaeoscyllium*
Carcharhiniformes	160	54.33	50.88				
†*Bavariscyllium*	4	9.22	6.32	**1.41E-33**			
Orectolobiformes	25	36.25	33.05	**1.53E-33**	**1.41E-33**		
†*Palaeoscyllium*	2	9.02	0	**1.32E-34**	5.01E-01	**1.32E-34**	

PART B. Phylogenetic analysis

To explore the phylogenetic relationships of †*Bavariscyllium* and †*Palaeoscyllium*, we incorporated them into a slightly amended and modified cladistic character-taxon matrix of Vullo et al. (2024). This resulted in a dataset comprising 212 morphological characters scored for 68 operational taxonomic units (OTUs). The character-taxon matrix was compiled in Mesquite 3.81 (Maddisson & Maddisson 2023) and analysed sing maximum parsimony in TNT v.1.6 (Goloboff & Morales 2023) under Goloboff et al.'s (2021) protocol for characters with logical dependencies.

Fossil material examined. †*Bavariscyllium tischlingeri* Thies, 2005 (JME SOS 4124 [holotype], LF 1436, SMF P 272, SMNS 96086); †*Palaeoscyllium formosum* Wagner, 1857 (SMNK-PAL 44950, SNSB-BSPG AS I 1365 [holotype], SNSB-BSPG AS I 589).

Character modifications and additions

Char. 33. Postorbital process: [0] forming part of the arcade, [1] arcade present bout notch separating postorbital process from supraotic shelf, [2] separated from arcade, [3] separated from arcade C bout notch separates postorbital process from supraotic shelf, [4] narrow filamentous, [5] reduced, [6] shelf-like laterally expanded, [7] shelf-like antero-ventrally expanded. Nishida (1990, char. 35), Brito & Seret (1996, char. 7), McEachran & Aschliman (2004, char. 32), Claeson et al. (2013, char. 12), Villalobos-Segura et al. (2022, char. 27), Vullo et al. (2024).
Remarks: We applied a multistate scoring for this character to account for the variation observed in the taxa included in the present analysis, implying homology among the different configurations of the postorbital process. Characters 35 and 58 of Vullo et al. (2024) are included as states within this multistate character. Although this could be considered a conglomerative character, the close relationship between the elements involved suggests a correlation in the development of the structures.

Char. 34. Triangular process: [0] absent, [1] present. McEachran et al. (1996, char. 29), Aschliman et al. (2012, char. 37), Villalobos-Segura et al. (2022, char. 29).
Remarks: The presence of a triangular process is defined as a function of the postorbital process and was therefore considered logically dependent on it by Vullo et al. (2024). In the present analysis, to account for the variation associated with the

postorbital process (char. 33), a multistate scoring was employed. Consequently, the logical relationship between the postorbital process and the triangular process was separated, and both characters were codified as independent entities.

Characters 60 and 99 of Vullo et al. (2024) present repeated information and were difficult to determine in most fossil species. Consequently, both characters were removed.

Char. 123. Scapular posterior process: [0] absent, [1] postero-dorsal triangular process of scapula, [2] both dorsal and ventral triangular processes present, [3] postero-ventral triangular process of scapula.
Remarks: This character is based on the observations made by da Silva et al. (2018) and includes char. 179 of Vullo et al. (2024).

Char. 151. Sup calcified vertebral centra: [0] absent, [1]. Vullo et al. (2024, char. 158).

Char. 152. Sub calcified vertebral centra (primary calcification): [0] restricted terminally, [0] well-developed across the whole body. Shirai (1992, char. 75), Shirai (1996, chars. 151, 152), Landemaine et al. (2018, char. 183).

Char. 153. Sub calcified vertebral centra (primary calcification): [0] absent, [1] endochordal radii radiating from the notochordal sheath, [2] with developed solid medialia and diagonal calcified lamellae, [3] compact mass. Shirai (1996, Char. 76), Landemaine et al. (2018, Char. 184), Jambura et al. (2023, Char. 130).

Char. 154. Cervical vertebra: [0] absent, [1] present, [2] fused with free vertebra reaching caudally to suprascapula, [3] fused, free vertebra present the entire length synarcual length, [4] fused, free vertebra reaching rostrally to suprascapula.
Remarks: With the modification of the codification for characters 151–153 in the present study, characters 154, 156 and 157 were treated as separate characters. Unfortunately, this resulted in an increased weight for the absence of vertebral centra (char. 151). While reductive coding may be a better option for characters 151–158, this is currently not feasible, as implementing such a coding strategy would generate more than 32 character states, which TNT cannot handle. Consequently, we chose to code them as separate characters with multiple states. Additionally, treating them as separate characters allows us to apply multistate scoring and incorporate a character

state tree (chars. 154 and 156), which helps us address the increased weight for the absent state.

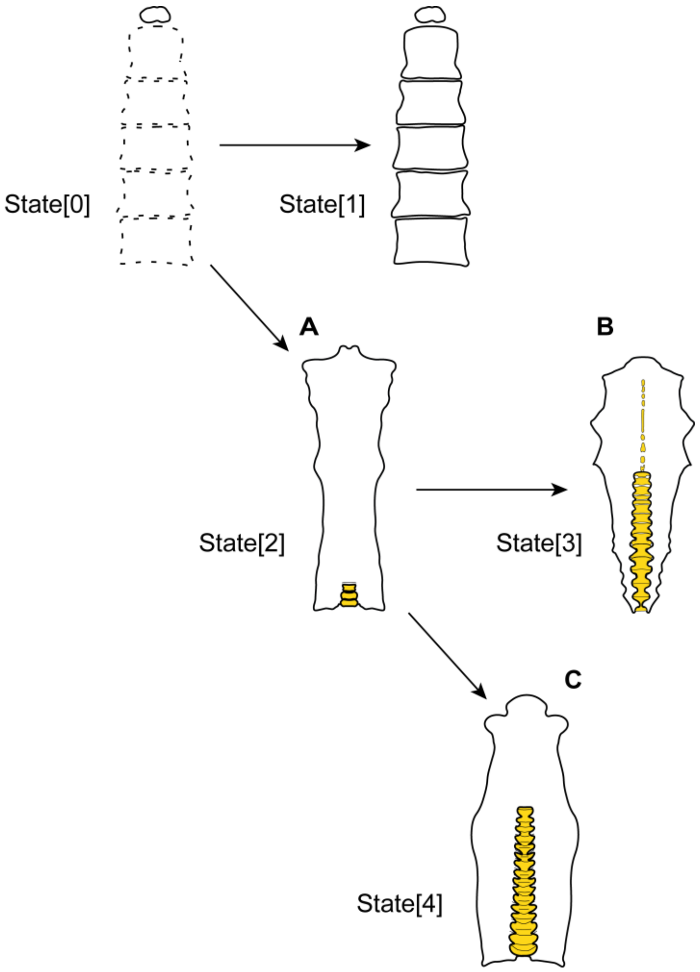

Supplementary Figure 4. Enforced path using the “cstree” command on TNT for state evolution on character 154. Arrows indicate the allowed transformation path starting from state [0], with each step increasing the state by one. Line drawings illustrate the states scored for this character. **A**, *Potamotrygon* (AMNH 38138); **B**, *Platyrhinoidis triseriata*; **C**, *Glaucostegus typus* (NHMUK 1967.2.11.3).

Characters 155–160 of Vullo et al. (2024) were modified. The initial codification aimed to follow a regionalization of the body and the modifications that the vertebra centra could present across these regions (e.g., fusions, synarcuals or expansion of the basiventral elements). However, to manage the number of characters states that TNT can handle (no more than 32), we kept the primary and secondary calcifications as separate characters (chars. 164–168). This decision created a conflict, as characters 164 and 166 coded for the absence or presence of these types of calcifications, providing redundant information compared to character 158, which are

codified for the presence or absence of calcified vertebral centra in Vullo et al. (2024). Consequently, in the present work, we aimed to avoid this redundancy by using reductive coding for the types of calcification present. Additionally, we employed multistate coding for character 153 due to uncertainty regarding the mechanisms that restrict or control the expression of secondary calcification. Specifically, we are unsure whether the genetic mechanisms for this type of calcification are present but silenced or absent.

Char. 158. Lateral stays: [0] absent, [1] dorsally directed and free of medial crest, [2] laterally directed and free of medial crest, [3] dorsally directed and fused to medial crest. Villalobos-Segura et al. (2019, char. 53); Villalobos-Segura et al. (2022, char. 52).

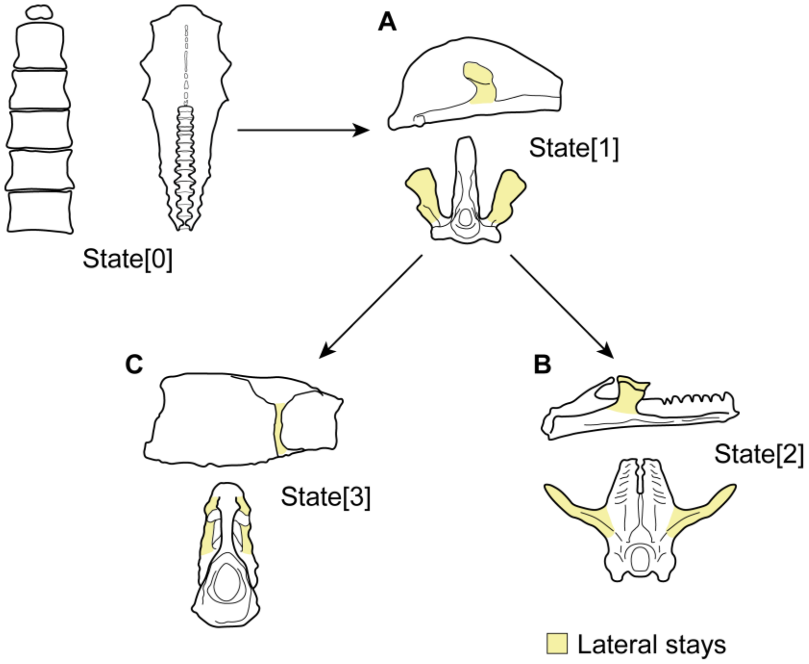

Supplementary Figure 5. Enforced path using the "cstree" command on TNT for state evolution on character 158. Arrows indicate the allowed transformation path starting from state [0], with each step increasing the state by one. **A**, *Rhina ancylostoma* (LACM 38117-38, https://sharksrays.org/, accessed on 20 Oct. 2023); **B**, *Torpedo fuscomaculata* (https://sharksrays.org/, accessed on 20 Oct. 2023); **C**, *Mobula munkiana* (SIO 85-34) (https://sharksrays.org/, accessed on 20 Oct. 2023).

Char. 211. Scapular process tip: [0] wide, [1] narrow. New.

Remarks: This character refers to the tip of the scapular process and whether this element fishing on an acute or a wide tip.

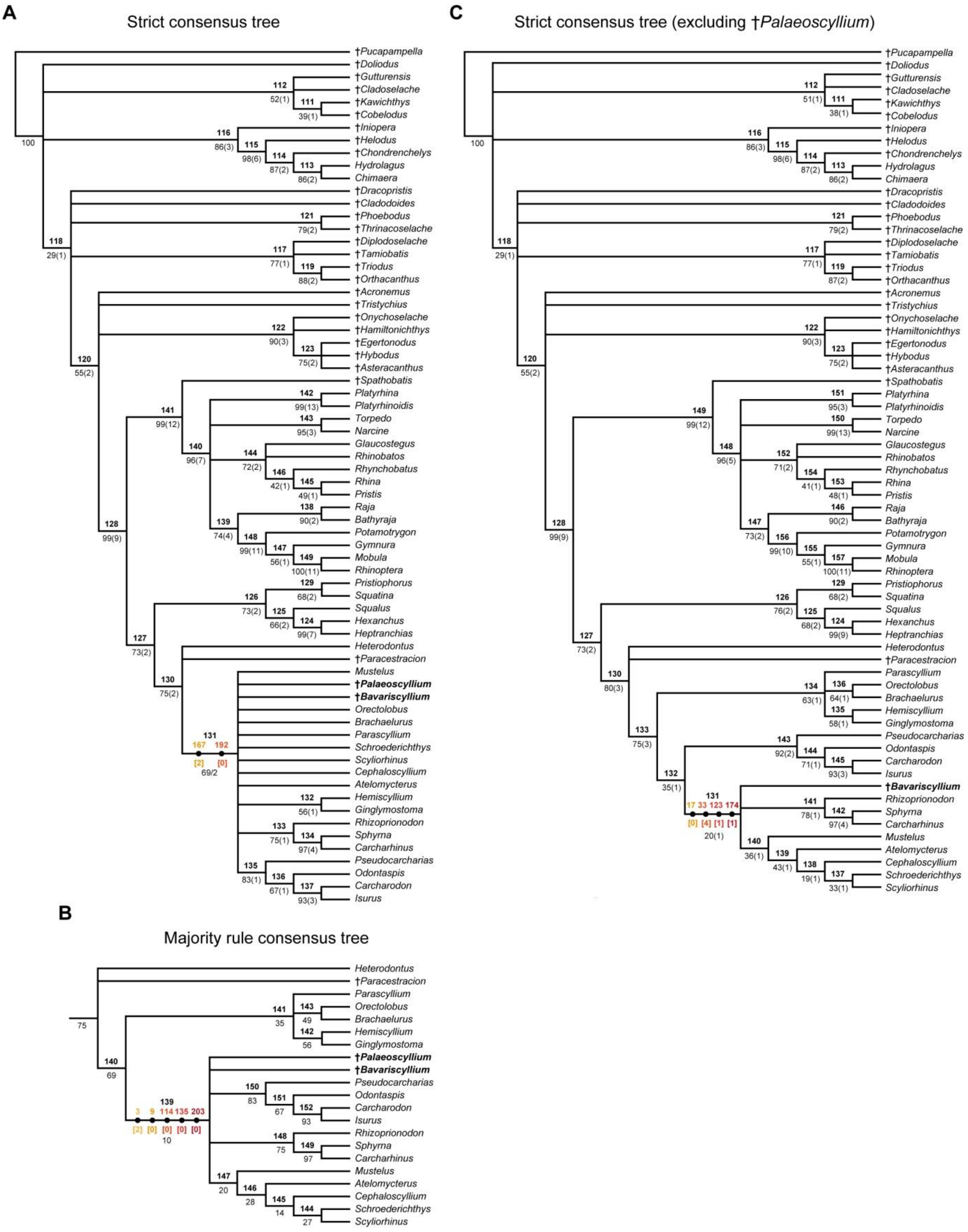

Supplementary Figure 6. Phylogenetic results from parsimony analysis. **A**, strict consensus tree based on complete dataset; **B**, majority rule consensus tree based on complete dataset showing the clade Galeomorphii; **C**, reduced strict consensus tree after removal of †*Palaeoscyllium.* Numbers next to nodes indicate the node number (bold), followed by bootstrap and jackknife values (with jackknife values in brackets). Characters supporting the

clades of interest are indicated by black dots. Character number is given above the point and character state is given below (in square brackets).

Characters supporting clades in tree A. Nodes marked with an asterisk (*) indicate characters that only support the given node in most, but not all, of the most parsimonious trees (MPTs).

Node 111*, chars.: 40[1]->[0], 45[0]->[1].

Node 112, chars.: 20[0]->[1], 118[0]->[1], 129[0]->[1], 132[1][3][5]->[0], 137[0]->[1], 141[1]->[0].

Node 113, chars.: 117[1]->[0], 132[1]->[2].

Node 114, chars.: 116[0]->[1], 149[0]->[1][2].

Node 115, chars: 33[0]->[5], 35[0]->[1], 50[0]->[1], 51[0]->[1], 52[0]->[1], 129[0]->[2], 210[0]->[1], 211[0]->[1].

Node 116, chars: 22[1]->[0], 46[0]->[2], 49[1]->[0], 55[1]->[0], 75[8]->[4], 78[0]->[1].

Node 117*, chars.: 11[2]->[4], 30[0]->[1], 41[0]->[1], 58[0]->[1], 116[0]->[1], 118[0]->[1], 120[0]->[1], 132[5]->[3], 165[1]->[0], 168[0]->[2].

Node 118, char.: 73[0]->[1].

Node 119, chars.: 163[0]->[1], 193[0]->[1].

Node 120, chars.: 42[0]->[1], 81[1]->[0], 91[0]->[1], 114[0]->[1].

Node 121, chars.: 8[0]->[1], 209[0]->[1].

Node 122, chars.: 33[0]->[7], 81[0]->[2], 185[0]->[3].

Node 123, char.: 207[0]->[1].

Node 124, chars.: 23[0]->[1], 39[0]->[1], 45[0]->[1], 54[1]->[0], 79[0]->[2], 80[0]->[1], 81[0]->[1], 91[1]->[5], 110[1]->[0], 117[0]->[1], 132[7]->[8], 151[5]->[1], 156[1]->[2], 160[1]->[0], 165[1]->[0], 167[1]->[5], 192[5]->[0], 204[0->[2].

Node 125, chars.: 7[0]->[1], 24[0]->[1], 48[0]->[1].

Node 126, chars.: 31[1]->[2], 75[5]->[6], 144[0]->[1].

Node 127, chars.: 131[1]->[2], 132[5]->[7], 154[0]->[1].

Node 128, chars.: 33[0]->[2], 69[0]->[1], 117[1]->[0], 151[0]->[1], 156[0]->[1], 157[0]->[1], 162[0]->[1].

Node 129, chars.: 35[3]->[4], 136[0]->[1], 167[1]->[3], 192[5]->[0].

Node 130, chars.: 3[0]->[3], 28[1]->[2].

Node 131*, chars.: 17[1]->[0], 33[2]->[4], 123[0]->[1], 174[0]->[1].

Node 132, chars.: 3[3]->[2], 114[1]->[0].

Node 133, chars.: 11[0]->[1], 13[1]->[0], 151[5]->[6][7], 167[1]->[2], 192[5]->[0].

Node 134, chars.: 54[1]->[0], 84[0]->[1], 108[0]->[1].

Node 135, chars.: 33[2]->[6], 125[0]->[1].

Node 136, chars.: 53[1]->[0], 85[0]->[1], 176[0]->[1].

Node 137, char.: 33[2->[3].

Node 138, char.: 33[4->[2].

Node 139, chars.: 27[0->[1], 177[0]->[1].

Node 140, char.: 123[1]->[2].

Node 141*, chars.: 19[0]->[1], 28[2]->[0], 44[0]->[1], 162[1]->[2].

Node 142, chars.: 118[0]->[1], 130[4]->[6], 131[2]->[3], 166[0]->[1].

Node 143, chars.: 30[0]->[2], 80[0]->[1], 81[0]->[1], 92[2]->[5].

Node 144, char.: 53[1]->[0],

Node 145, chars.: 118[0]->[1], 166[0]->[1], 168[1]->[0].

Node 146, chars.: 65[1]->[0], 140[0]->[1], 190[0]->[1].

Node 147, chars.: 28[1]->[2], 104[0]->[1], 162[1]->[2], 166[0]->[1], 174[0]->[1], 178[0->[1].

Node 148, chars.: 124[0]->[1][2], 131[1]->[3], 132[5]->[9], 158[0]->[1][2], 160[1]->[2], 192[5]->[0].

Node 149, chars.: 23[0]->[2], 31[1]->[0], 54[1]->[0], 75[5]->[0], 91[1]->[2], 92[2]->[0], 98[0]->[1], 99[1]->[4], 109[0]->[1], 118[0]->[1], 136[0]->[1], 154[0]->[2], 156[1]->[3], 168[1]->[2].

Node 150, chars.: 28[1]->[0], 33[2]->[5], 89[0]->[1], 96[0]->[1], 111[0]->[1], 112[13]->[0], 121[0]->[1], 127[1][2][3]->[0], 140[0]->[1], 143[0]->[1], 184[0]->[1], 189[1]->[0].

Node 151, chars.: 13[1]->[3], 16[0]->[1], 164[0]->[1].

Node 152, chars.: 23[2]->[6][7], 154[2]->[4].

Node 153, chars.: 25[1]->[0], 154[4]->[2], 177[0]->[1].

Node 154, chars.: 142[1]->[0], 168[2]->[1].

Node 155, chars.: 65[1]->[0], 138[0]->[1], 189[1]->[0].

Node 156, chars.: 2[0]->[1], 13[1]->[0], 15[0]->[1], 25[1]->[2], 89[0]->[1], 90[0]->[1], 155[0]->[1], 165[1]->[2], 179[0]->[1], 183[0]->[1], 198[0]->[1].

Node 157, chars.: 2[1]->[2], 18[0]->[1], 28[2]->[1], 33[2][8]->[6], 74[0]->[1], 83[0]->[1], 99[2][4][5]->[0], 122[0]->[1].

Node 158, chars.: 165[2]->[0], 172[0]->[1].

Characters supporting clades in tree B. Nodes marked with an asterisk (*) indicate characters that only support the given node in most, but not all, of the most parsimonious trees (MPTs).

Node 111*, chars.: 40[1]->[0], 45[0]->[1].

Node 112, chars.: 20[0]->[1], 118[0]->[1], 129[0]->[1], 132[1][3][5]->[0], 137[0]->[1], 141[1]->[0].

Node 113, chars.: 117[1]->[0], 132[1]->[2].

Node 114, chars.: 116[0]->[1], 149[0]->[1][2].

Node 115, chars: 33[0]->[5], 35[0]->[1], 50[0]->[1], 51[0]->[1], 52[0]->[1], 129[0]->[2], 210[0]->[1], 211[0]->[1].

Node 116, chars: 22[1]->[0], 46[0]->[2], 49[1]->[0], 55[1]->[0], 75[8]->[4], 78[0]->[1].

Node 117*, chars.: 11[2]->[4], 30[0]->[1], 41[0]->[1], 58[0]->[1], 116[0]->[1], 118[0]->[1], 120[0]->[1], 132[5]->[3], 165[1]->[0], 168[0]->[2].

Node 118, char.: 73[0]-> [1].

Node 119, chars.: 163[0]->[1], 193[0]->[1].

Node 120, chars.: 42[0]->[1], 81[1]->[0], 91[0]->[1], 114[0]->[1].

Node 121, chars.: 8[0]->[1], 209[0]->[1].

Node 122, chars.: 33[0]->[7], 81[0]->[2], 185[0]->[3].

Node 123, char.: 207[0]->[1].

Node 124, chars.: 23[0]->[1], 39[0]->[1], 45[0]->[1], 54[1]->[0], 79[0]->[2], 80[0]->[1], 81[0]->[1], 91[1]->[5], 110[1]->[0], 117[0]->[1], 132[7]->[8], 151[5]->[1], 156[1]->[2], 160[1]->[0], 165[1]->[0], 167[1]->[5], 192[5]->[0], 204[0->[2].

Node 125, chars.: 7[0]->[1], 24[0]->[1], 48[0]->[1].

Node 126, chars.: 31[1]->[2], 75[5]->[6], 144[0]->[1].

Node 127, chars.: 131[1]->[2], 132[5]->[7], 154[0]->[1].

Node 128, chars.: 33[0]->[2], 69[0]->[1], 117[1]->[0], 151[0]->[1], 156[0]->[1], 157[0]->[1], 162[0]->[1].

Node 129, chars.: 35[3]->[4], 136[0]->[1], 167[1]->[3], 192[5]->[0].

Node 130, chars.: 3[0]->[3], 28[1]->[2].

Node 131, chars.: 167[1]-->[2], 192[5]->[0].

Node 132*, chars.: 33[2]->[6], 125[0]->[1], 132[7]->[0].

Node 133*, chars.: 19[0]->[1], 28[2]->[0], 44[0]->[1], 162[1]->[2].

Node 134, chars.: 118[0]->[1], 130[4]->[6], 131[2]->[3], 165[0]->[1].

Node 135*, chars.: 30[0]->[2], 44[0]->[1], 80[0]->[1], 81[0]->[1], 92[2]->[5], 162[1]->[2].

Node 136, char.: 53[1]->[0].

Node 137, chars.: 118[0]->[1], 166[0]->[1], 168[1]->[0].

Node 138, chars.: 65[1]->[0], 140[0]->[1], 190[0]->[1].

Node 139, chars.: 28[1]->[2], 104[0]->[1], 162[1]->[2], 166[0]->[1], 176[0]->[1], 178[0]->[1].

Node 140, chars.: 124[0]->[1][2], 131[1]->[3], 132[5]->[9], 158[0]->[1][2], 160[1]->[2], 192[5]->[0].

Node 141, chars.: 23[0]->[2], 31[1]->[0], 54[1]->[0], 75[5]->[0], 91[1]->[2], 92[2]->[0], 98[0]->[1], 99[1]->[4], 109[0]->[1], 118[0->[1], 136[0]->[1], 154[0]->[2], 156[1]->[3], 168[1]->[2].

Node 142, chars.: 28[1]->[0], 33[2]->[5], 89[0]->[1], 96[0]->[1], 111[0]->[1], 112[1][3->[0], 121[0]->[1], 127[1][2][3]->[0], 140[0]->[1], 143[0]->[1], 184[0]->[1], 189[1]->[0].

Node 143, chars.: 13[1]->[3], 16[0]->[1], 164[0]->[1].

Node 144, chars.: 23[2]->[6][7], 154[2]->[4].

Node 145, chars.: 25[1]->[0], 154[4]->[2], 177[0]->[1].

Node 146, chars.: 142[1]->[0], 168[2]->[1].

Node 147, chars.: 65[1]->[0], 138[0]->[1], 189[1]->[0].

Node 148, chars.: 2[0]->[1], 13[1]->[0], 15[0]->[1], 25[1]->[2], 89[0]->[1], 90[0]->[1], 155[0]->[1], 165[1]->[2], 179[0]->[1], 183[0]->[1], 198[0]->[1].

Node 149, chars.: 2[1]->[2], 18[0]->[1], 28[2]->[1], 33[2][8]->[6], 74[0->[1], 83[0]->[1], 99[2][4][5]->[0], 122[0]->[1], 158[1]->[3], 165[2]->[0], 172[0]->[1].

Characters supporting clades in tree C. Nodes marked with an asterisk (*) indicate characters that only support the given node in most, but not all, of the most parsimonious trees (MPTs).

Node 139, chars.: 3[3]->[2], 9[1]->[0], 114[1]->[0], 135[1]->[0], 203[2]->[0].

Node 140, chars.: 11[0]->[1], 13[1]->[0], 45[0]->[1], 151[5]->[7], 167[1]->[2], 192[5]->[0].

Node 141, chars.: 27[0]->[1], 54[1]->[0], 84[0->[1], 101[1]->[2], 108[0]->[1].

Node 142, chars.: 33[2]->[6], 125[0]->[1], 132[7]->[0], 151[7]->[6].

Node 143, chars.: 53[1]->[0], 85[0]->[1], 176[0]->[1].

Node 144, char.: 33[2]->[3].

Node 146, chars.: 27[0]->[1], 177[0]->[1],

Node 147, chars.: 123[0][1]->2, 146[2]->[1].

Node 148, chars.: 19[0->[1], 28[2]->[0], 44[0]->[1], 146[2]->[3], 162[1]->[2].

Node 149, chars.: 118[0]->[1], 130[4]->[6], 131[2]->[3], 166[0]->[1].

Node 150, chars.: 30[0]->[2], 44[0]->[1], 80[0]->[1], 81[0]->[1], 92[2]->[5], 151[7]->[6], 162[1]->[2], 203[0]->[1].

Node 151, chars.: 53[1]->[0], 212[1]->[0].

Node 152, chars.: 118[0]->[1], 166[0]->[1], 168[1]->[0].

Node 153, chars.: 43[0]->[1], 65[1]->[0], 140[0]->[1], 190[0]->[1].

Node 154, chars.: 28[1]->[2], 104[0]->[1], 142[1]->[0], 162[1]->[2], 166[0]->[1], 176[0]->[1], 178[0]->[1].

Node 155, chars.: 124[0]->[1], 130[4]->[6], 131[1]->[3], 132[5]->[9], 158[0]->[1], 160[1]->[2], 192[5]->[0].

Node 156, chars.: 23[0]->[2], 31[1]->[0], 54[1]->[0], 75[5]->[0], 91[1]->[2], 92[2]->[0], 98[0]->[1], 99[1]->[4], 109[0]->[1], 118[0]->[1], 136[0]->[1], 142[0]->[1], 154[0]->[2], 156[1]->[3], 168[1]->[2].

Node 157, chars.: 23[2]->[3], 28[1]->[0], 33[2]->[5], 89[0]->[1], 96[0]->[1], 111[0]->[1], 112[1]->[0], 117[0]->[1], 121[0]->[1], 127[1][2][3]->[0], 130[6]->[7], 140[0]->[1], 143[0]->[1], 158[1]->[2], 184[0]->[1], 189[1]->[0].

Node 158, chars.: 13[1]->[3], 16[0]->[1], 99[4]->[0], 112[1]->[3], 158[1]->[2], 164[0]->[1].

Node 159, chars.: 35[3]->[5], 43[0]->[1], 124[1]->[2], 159[0]->[1], 202[0]->[1].

Node 160, chars.: 18[0]->[1], 23[2]->[6][7], 137[0]->[1], 154[2]->[4], 177[1]->[0].

Node 161, chars.: 25[1]->[0], 154[4]->[2], 177[0]->[1].

Node 162, chars.: 142[1]->[0], 168[2]->[1].

Node 163, char.: 33[2]->[3].

Node 164, chars.: 65[1]->[0], 138[0]->[1], 189[1]->[0].

Node 165, chars.: 2[0]->[1], 13[1]->[0], 15[0]->[1], 25[1]->[2], 35[3]->[5], 89[0]->[1], 90[0->[1], 124[1]->[3], 155[0]->[1], 165[1]->[2], 179[0]->[1], 183[0]->[1], 198[0]->[1].

Node 166, chars.: 2[1]->[2], 18[0]->[1], 28[2]->[1], 33[2][8]->[6], 74[0]->[1], 83[0]->[1], 99[2][4][5]->[0], 122[0]->[1], 158[1]->[3], 165[2]->[0], 172[0]->[1]

PART C. Additional information

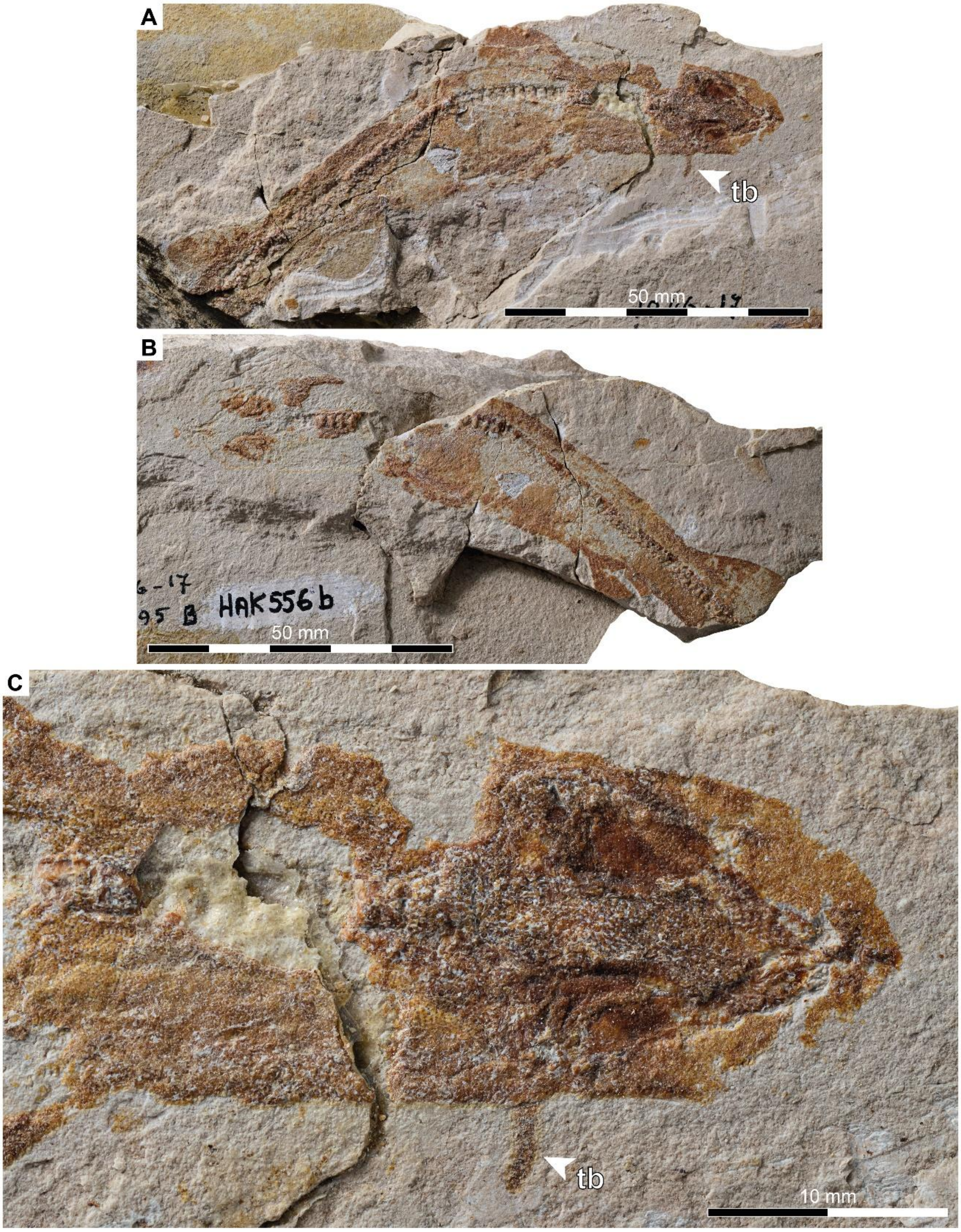

Supplementary Figure 7. Holotype and only known specimen of †*Pararhincodon lehmani* Cappetta, 1980 (MNHN FHAK556a, b) from the Cenomanian of Haqel, Lebanon. **A**, **B**, complete specimen preserved in (**A**) part (MNHN FHAK556a) and (**B**) counterpart (MNHN FHAK556b); **C**, close-up view of head preserved in MNHN FHAK556b. Anatomical abbreviation: tb, throat barbel.

Supplementary References

Aschliman, N.C., Claeson, K.M., & McEachran, J.D. 2012. Phylogeny of Batoidea; pp. 57–97 in Carrier, C., Musick, J.A., & Heithaus, M.R. (eds.), Biology of Sharks and their Relatives. CRC Press.

Brito, P.M., & Seret, B. 1996. The new genus *Iansan* (Chondrichthyes, Rhinobatoidea) from the Early Cretaceous of Brazil and its phylogenetic relationships; pp. 47–63 in Arratia, G., & Viohl, G. (eds.), Mesozoic Fshes. Systematics and Paleoecology. Verlag Dr. Friedrich Pfeil.

Cappetta, H. 1980. Les Sélaciens du Crétacé supérieur du Liban. I. Requins. Palaeontographica, Abteilung A, 168(1–4), 69–148.

Claeson, K.M., Underwood, C.J., & Ward, D.J. 2013. †*Tingitanius tenuimandibulus*, a new platyrhinid batoid from the Turonian (Cretaceous) of Morocco and the Cretaceous radiation of the Platyrhinidae. Journal of Vertebrate Paleontology, 33(5), 1019–1036.

da Silva, J.P.C.B., Vaz, D.F., & de Carvalho, M.R. 2018. Phylogenetic inferences on the systematics of squaliform sharks based on elasmobranch scapular morphology (Chondrichthyes: Elasmobranchii). Zoological Journal of the Linnean Society, 182(3), 614-630.

Ebert, D.A., Dando, M., & Fowler, S. 2021. Sharks of the world: a complete guide. Princeton University Press.

Goloboff, P.A., & Morales, M.E. 2023. TNT version 1.6, with a graphical interface for MacOS and Linux, including new routines in parallel. Cladistics, 39(2), 144–153.

Goloboff, P.A., De Laet, J., Ríos-Tamayo, D., & Szumik, C.A. 2021. A reconsideration of inapplicable characters, and an approximation with step-matrix recoding. Cladistics, 37(5), 596–629.

Jambura, P.L., Villalobos-Segura, E., Türtscher, J., Begat, A., Staggl, M.A., Stumpf, S., Kindlimann, R., Klug, S., Lacombat, F., Pohl, B., Maisey, G.J., Naylor, G.J.P., & Kriwet, J. 2023. Systematics and phylogenetic interrelationships of

the enigmatic Late Jurassic shark *Protospinax annectans* Woodward, 1918 with comments on the shark–Ray sister group relationship. Diversity, 15, 311.

Kriwet, J., & Klug, S. 2004. Late Jurassic selachians (Chondrichthyes, Elasmobranchii) from southern Germany: re-evaluation on taxonomy and diversity. Zitteliana, Reihe A, 44, 67–95.

Landemaine, O., Thies, D., & Waschkewitz, J. 2018. The Late Jurassic shark *Palaeocarcharias* (Elasmobranchii, Selachimorpha) – functional morphology of teeth, dermal cephalic lobes and phylogenetic position. Palaeontographica Abteilung A, 312, 103–165.

Leidner, A., & Thies, D. 1999. Placoid scales and oral teeth of Late Jurassic elasmobranchs from Europe; pp. 29–40 in Arratia, G., & Schultze, H.-P. (eds.), Mesozoic Fishes 2. Systematics and Fossil Record. Verlag Dr. Friedrich Pfeil.

Maddison, W.P., & Maddison, D.R. 2023. Mesquite: a modular system for evolutionary analysis. Version 3.81.

McEachran, J.D., & Aschliman, N. 2004. Phylogeny of Batoidea; pp. 79–109 in Carrier, C., Musick, J.A., & Heithaus, M.R. (eds.), Biology of Sharks and their Relatives. CRC Press.

McEachran, J.D., Dunn, K.A., & Miyake, T. 1996. Interrelationships of the batoid fishes (Chondrichthyes: Batoidea); pp. 63–83 in Stiassny, M.L.J., Parenti, L.R., & Johnson, G.D. (eds.), Interrelationships of Fishes. Atlantic Press.

Nishida, K. 1990. Phylogeny of the Suborder Myliobatidoidei. Memoirs of the Faculty of Fisheries, Hokkaido University, 37, 1–108.

Shirai, S. 1992. Squalean Phylogeny: A new framework of “squaloid” sharks and related taxa. Hokkaido University Press, 151 pp.

Shirai, S. 1996. Phylogenetic interrelationships of neoselachians (Chondrichthyes: Euselachii); pp. 9–34 in Stiassny, M.L.J., Parenti, L.R., & Johnson, G.D. (eds.), Interrelationships of Fishes. Atlantic Press.

Thies, D. 2005. A catshark (Neoselachii, Carcharhiniformes, Scyliorhinidae) from the Late Jurassic of Germany. Paläontologische Zeitschrift, 79(3), 339–348.

Thies, D., & Leidner, A. 2011. Sharks and guitarfishes (Elasmobranchii) from the Late Jurassic of Europe. Palaeodiversity, 4, 63–184.

Villalobos-Segura, E., Underwood, C.J., Ward, D.J., & Claeson, K.M. 2019. The first three-dimensional fossils of Cretaceous sclerorhynchid sawfish: *Asflapristis cristadentis* gen. et sp. nov., and implications for the phylogenetic relations of the Sclerorhynchoidei (Chondrichthyes). Journal of Systematic Palaeontology, 17(21), 1847–1870.

Villalobos-Segura, E., Marramà, G., Carnevale, G., Claeson, K.M., Underwood, C.J., Naylor, G.J.P., & Kriwet, J. 2022. The phylogeny of rays and skates (Chondrichthyes: Elasmobranchii) based on morphological characters revisited. Diversity, 14, 1–65.

Vullo, R., Villalobos-Segura, E., Amadori, M., Kriwet, J., Frey, E., González González, M.A., Padilla Gutiérrez, J.M., Ifrim, C., Stinnesbeck, E.S., & Stinnesbeck, W. 2024. Exceptionally preserved shark fossils from Mexico elucidate the long-standing enigma of the Cretaceous elasmobranch *Ptychodus*. Proceedings of the Royal Society B, 291, 20240262.

Wagner, J.A. 1857. Charakteristik neuer Arten von Knorpelfischen aus den lithographischen Schiefern der Umgegend von Solnhofen. Gelehrte Anzeigen der Königlichen Bayerischen Akademie der Wissenschaften, 44, 288–293.
