# Supplementary material for: Reappraisal of the extinct barbelthroat shark †Bavariscyllium and the nebulous origin of carcharhiniform galeomorphs
Source: Commun Biol. 2026 Feb 17;9:158. doi: 10.1038/s42003-025-09272-5 (PMC12913886; doi:10.1038/s42003-025-09272-5)
Supplement: Supplementary file 2 — Description of Additional Supplementary Files [file 42003_2025_9272_MOESM2_ESM.pdf]

Description of Additional Supplementary Files

File name: Supplementary Data 1

Description: XLSX file containing all species and corresponding linear measurements used in the morphometric analysis.

File name: Supplementary Data 2

Description: TXT file containing character-taxon matrix used in the phylogenetic analysis.
