# Supplementary material for: Reappraisal of the extinct barbelthroat shark †Bavariscyllium and the nebulous origin of carcharhiniform galeomorphs
Source: Commun Biol. 2026 Feb 17;9:158. doi: 10.1038/s42003-025-09272-5 (PMC12913886; doi:10.1038/s42003-025-09272-5)
Supplement: Supplementary file 5 — Reporting Summary [file 42003_2025_9272_MOESM5_ESM.pdf]

nature portfolio

Corresponding author(s): Sebastian Stumpf, Jürgen Kriwet

Last updated by author(s): May 12, 2025

Reporting Summary

Nature Portfolio wishes to improve the reproducibility of the work that we publish. This form provides structure for consistency and transparency in reporting. For further information on Nature Portfolio policies, see our Editorial Policies and the Editorial Policy Checklist.

Statistics

For all statistical analyses, confirm that the following items are present in the figure legend, table legend, main text, or Methods section.

n/a	Confirmed	
☐	☒	The exact sample size (*n*) for each experimental group/condition, given as a discrete number and unit of measurement
☐	☒	A statement on whether measurements were taken from distinct samples or whether the same sample was measured repeatedly
☐	☒	The statistical test(s) used AND whether they are one- or two-sided<br>*Only common tests should be described solely by name; describe more complex techniques in the Methods section.*
☐	☒	A description of all covariates tested
☐	☒	A description of any assumptions or corrections, such as tests of normality and adjustment for multiple comparisons
☐	☒	A full description of the statistical parameters including central tendency (e.g. means) or other basic estimates (e.g. regression coefficient) AND variation (e.g. standard deviation) or associated estimates of uncertainty (e.g. confidence intervals)
☐	☒	For null hypothesis testing, the test statistic (e.g. F, t, r) with confidence intervals, effect sizes, degrees of freedom and P value noted<br>*Give P values as exact values whenever suitable.*
☒	☐	For Bayesian analysis, information on the choice of priors and Markov chain Monte Carlo settings
☒	☐	For hierarchical and complex designs, identification of the appropriate level for tests and full reporting of outcomes
☒	☐	Estimates of effect sizes (e.g. Cohen's *d*, Pearson's *r*), indicating how they were calculated

Our web collection on statistics for biologists contains articles on many of the points above.

Software and code

Policy information about availability of computer code

Data collection	ImageJ v1.53t, https://imagej.net/ij/index.html; Mesquite 3.81, https://www.mesquiteproject.org/
Data analysis	R software, https://www.r-project.org/; TNT v.1.6, https://www.lillo.org.ar/phylogeny/tnt/. All relevant R packages are provided in the paper.

For manuscripts utilizing custom algorithms or software that are central to the research but not yet described in published literature, software must be made available to editors and reviewers. We strongly encourage code deposition in a community repository (e.g. GitHub). See the Nature Portfolio guidelines for submitting code & software for further information.

Data

Policy information about availability of data

All manuscripts must include a data availability statement. This statement should provide the following information, where applicable:

- Accession codes, unique identifiers, or web links for publicly available datasets
- A description of any restrictions on data availability
- For clinical datasets or third party data, please ensure that the statement adheres to our policy

All data supporting the findings of this study are provided in the paper and its Supplementary Information. The fossil material presented in this study is housed in publicly accessible collections, with details available in the paper and its Supplementary Information.

Research involving human participants, their data, or biological material

Policy information about studies with human participants or human data. See also policy information about sex, gender (identity/presentation), and sexual orientation and race, ethnicity and racism.

Reporting on sex and gender	Not applicable
Reporting on race, ethnicity, or other socially relevant groupings	Not applicable
Population characteristics	Not applicable
Recruitment	Not applicable
Ethics oversight	Not applicable

Note that full information on the approval of the study protocol must also be provided in the manuscript.

Field-specific reporting

Please select the one below that is the best fit for your research. If you are not sure, read the appropriate sections before making your selection.

☒ Life sciences ☐ Behavioural & social sciences ☐ Ecological, evolutionary & environmental sciences

For a reference copy of the document with all sections, see nature.com/documents/nr-reporting-summary-flat.pdf

Life sciences study design

All studies must disclose on these points even when the disclosure is negative.

Sample size	The sample size for this study was determined based on the availability of suitable fossil and extant specimens, as no formal statistical sample size calculation was performed. The fossil dataset included four holomorphic (i.e., virtually complete articulated) specimens of †Bavariscyllium tischlingeri and two holomorphic specimens of †Palaeoscyllium formosum. These specimens were selected due to their completeness and the availability of measurable features. For extant species, morphometric data were obtained through illustrations from Ebert's et al. (2021) Sharks of the World: A Complete Guide for 160 carcharhiniform and 25 orectolobiform species, representing five families of phenotypically similar sharks: Scyliorhinidae, Atelomycteridae, Pentanchidae, Parascylliidae, and Hemiscylliidae. These families were chosen based on their phenotypic similarity to the fossil taxa and their relevance to the study's objectives. The rationale for the chosen sample size was to maximize the representation of both fossil and extant taxa while ensuring sufficient data to conduct robust statistical analyses. To account for differences in sample size between groups, a bootstrap approach with 100 iterations was applied during the analysis of morphological disparity.
Data exclusions	Data exclusions were applied to ensure the reliability and consistency of the analyses. Measurements were expressed as percentages of the total length (TL) of each specimen. Consequently, fossil specimens lacking total length data could not be included in the study. For instance, †Corysodon cirinensis and †Pararhincodon lehmani were excluded due to the absence of suitable fossil material. Additionally, missing values for one specimen of †Bavariscyllium tischlingeri (SMNS 96086), where dorsoventral preservation prevented all measurements from being taken, were imputed using a regularized iterative principal component analysis (PCA) algorithm with the imputePCA function from the R package missMDA.
Replication	To verify the reproducibility of the experimental findings, several measures were implemented throughout the study. Measurements were taken using ImageJ v1.53t, with each measurement repeated three times and averaged to minimize potential errors and ensure consistency. In addition, the dataset was subjected to rigorous statistical tests. The use of multiple statistical methods, such as PCA, ANOVA, and Kruskal-Wallis tests, provided robust checks on the findings. The analysis of morphological disparity employed a bootstrap approach with 100 iterations to account for differences in sample size, which allowed for more reliable estimates and increased the reproducibility of the results.
Randomization	Samples were allocated into experimental groups based on taxonomic identity and specimen completeness rather than through random allocation. Allocation was driven by the availability of suitable fossil material and the focus on comparing phenotypically similar groups (see above). Covariates were controlled by standardizing measurements as percentages of total length (TL) for all specimens, which ensured consistency across the groups. Random allocation was not relevant to this study because the focus was on taxonomic and phenotypic comparisons between predefined groups.
Blinding	The focus of the study was on comparing predefined groups of fossil and extant shark species, so blinding was not deemed relevant for the specific objectives of this study.

Reporting for specific materials, systems and methods

We require information from authors about some types of materials, experimental systems and methods used in many studies. Here, indicate whether each material, system or method listed is relevant to your study. If you are not sure if a list item applies to your research, read the appropriate section before selecting a response.

Materials & experimental systems

n/a	Involved in the study
☒	☐ Antibodies
☒	☐ Eukaryotic cell lines
☐	☒ Palaeontology and archaeology
☒	☐ Animals and other organisms
☒	☐ Clinical data
☒	☐ Dual use research of concern
☒	☐ Plants

Methods

n/a	Involved in the study
☒	☐ ChIP-seq
☒	☐ Flow cytometry
☒	☐ MRI-based neuroimaging

Palaeontology and Archaeology

Specimen provenance	The articulated specimens discussed in the main text are from the Upper Jurassic Solnhofen Archipelago in southern Germany, renowned as a classic example of a Konservat-Lagerstätte due to its exceptional preservation. Additionally, 17 isolated teeth are described from early Kimmeridgian marls near Mahlstetten, also in southern Germany.
Specimen deposition	All the fossil material presented in this study is held in publicly accessible collections at the Jura-Museum Eichstätt, Eichstätt, Germany; Lauer Foundation for Paleontology, Science & Education, Wheaton, Illinois, USA; Staatliches Museum für Naturkunde Karlsruhe, Germany; Senckenberg Naturmuseum, Frankfurt, Germany; Staatliches Museum für Naturkunde Stuttgart, Germany; Bayerische Staatssammlung für Paläontologie und Geologie, Munich, Germany.
Dating methods	No new dates are provided.

☐ Tick this box to confirm that the raw and calibrated dates are available in the paper or in Supplementary Information.

Ethics oversight	No ethical approval or guidance was required for this study as it involved the analysis of fossil specimens housed in publicly accessible collections.

Note that full information on the approval of the study protocol must also be provided in the manuscript.

Plants

Seed stocks	Not applicable
Novel plant genotypes	Not applicable
Authentication	Not applicable
